# Culture-Facilitated Comparative Genomics of the Facultative Symbiont *Hamiltonella defensa*

**DOI:** 10.1093/gbe/evy036

**Published:** 2018-02-14

**Authors:** Germain Chevignon, Bret M Boyd, Jayce W Brandt, Kerry M Oliver, Michael R Strand

**Affiliations:** Department of Entomology, University of Georgia

**Keywords:** insect, aphid, bacteria, comparative genomics, parasitoid, defense

## Abstract

Many insects host facultative, bacterial symbionts that confer conditional fitness benefits to their hosts. *Hamiltonella defensa* is a common facultative symbiont of aphids that provides protection against parasitoid wasps. Protection levels vary among strains of *H. defensa* that are also differentially infected by bacteriophages named APSEs. However, little is known about trait variation among strains because only one isolate has been fully sequenced. Generating complete genomes for facultative symbionts is hindered by relatively large genome sizes but low abundances in hosts like aphids that are very small. Here, we took advantage of methods for culturing *H. defensa* outside of aphids to generate complete genomes and transcriptome data for four strains of *H. defensa* from the pea aphid *Acyrthosiphon pisum*. Chosen strains also spanned the breadth of the *H. defensa* phylogeny and differed in strength of protection conferred against parasitoids. Results indicated that strains shared most genes with roles in nutrient acquisition, metabolism, and essential housekeeping functions. In contrast, the inventory of mobile genetic elements varied substantially, which generated strain specific differences in gene content and genome architecture. In some cases, specific traits correlated with differences in protection against parasitoids, but in others high variation between strains obscured identification of traits with likely roles in defense. Transcriptome data generated continuous distributions to genome assemblies with some genes that were highly expressed and others that were not. Single molecule real-time sequencing further identified differences in DNA methylation patterns and restriction modification systems that provide defense against phage infection.

## Introduction

Heritable microbial symbionts are important drivers of evolution in many multicellular organisms (Moran et al. 2008). In insects, heritable bacterial symbionts are subdivided into obligate (=primary) species, which persist by providing nutrients essential for host survival, and facultative (=secondary) species, which persist by providing conditional benefits or manipulating the reproduction of hosts (Moran et al. 2008; Oliver and Martinez 2014). Aphids (Hemiptera: Aphidoidea) are an economically important group of phloem feeding insects that have emerged as models for the study of heritable symbiosis. Almost all aphid species harbor *Buchnera aphidicola*, which is a specialized, obligate symbiont that resides in host cells called bacteriocytes (Douglas 1998). Aphids may be additionally infected by one or more species of facultative symbionts (Oliver et al. 2010)*.* Although some aphid facultative symbionts mediate interactions with food plants (Tsuchida et al. 2004; Wagner et al. 2015), many more protect aphids against biotic and abiotic threats (Oliver and Martinez 2014). The γ-proteobacterium *Hamiltonella defensa* is among the most common facultative symbionts, occurring in 34% of sampled aphid species (Henry et al. 2015; Zytynska and Weisser 2016), and conditionally enhancing the fitness of at least three by protecting them against parasitoid wasps (Oliver et al. 2003; Schmid et al. 2012; Asplen et al. 2014).

Typing diagnostics indicate many strains of *H. defensa* exist (Degnan and Moran 2008b; Ferrari et al. 2012; Henry et al. 2013; Russell et al. 2013), which are usually persistently infected by a Podovirus-like bacteriophage named APSE (van der Wilk et al. 1999; Sandström et al. 2001; Moran et al. 2005). Several APSEs are also recognized on the basis of a variable domain in the viral genome that encodes predicted eukaryotic toxin genes (Degnan and Moran 2008a). *H. defensa* and proviral APSEs are vertically transmitted at high rates but spontaneous APSE loss in some *H. defensa* strains results in ablated protection against parasitoids (Oliver et al. 2009; Weldon et al. 2013). Strains of *H. defensa* and/or APSE further vary in the levels of protection they confer against different species or genotypes of parasitoids (Oliver et al. 2005; Rouchet and Vorburger 2012; Asplen et al. 2014; Cayetano and Vorburger 2015; McLean and Godfray 2015; Martinez et al. 2016; Hopper et al. 2017; Martinez AJ et al. 2017), while also differentially affecting parasitoid competitive interactions in the same host (Oliver et al. 2012; McLean and Godfray 2015; Kraft et al. 2017).

*H. defensa* and APSE are primarily maternally transmitted but horizontal transfer events have also influenced distribution. Transinfection experiments show that some *H. defensa* strains readily move among clones of the pea aphid, *Acyrthosiphon pisum*, and between aphid species (Oliver et al. 2005) while some APSE variants move between *H. defensa* strains (Brandt et al. 2017). Phylogenetic studies support a history of horizontal transfer of *H. defensa* within and among aphid species and its presence in psyllids and whiteflies indicates lateral movement among sternorrhynchan Hemiptera (Sandström et al. 2001; Russell et al. 2003; Oliver et al. 2010; Henry et al. 2013, 2015). APSE-derived prophage islands are also present in the genomes of several *Arsenophonus* strains (Duron 2014), an endosymbiont present in diverse insect groups, although publicly available assemblies suggest these proviral genomes are not fully intact. Thus, APSEs may have broader host ranges than *H. defensa*.

Outside of aphids, *H. defensa* is best-studied in the MED (Q) and MEAM1 (B) biotypes of the *Bemisia tabaci* (Hemiptera: Aleyrodidae) cryptic species complex (Rao et al. 2015). No evidence supports a role for *H. defensa* in protecting whiteflies against parasitoids, and the APSEs linked to protection in aphids appear inactivated in *B. tabaci* strains (Rollat-Farnier et al. 2015). However, *H. defensa* may play roles in modulating interactions between *B. tabaci* (MED) and plant defenses (Su et al. 2015) or affecting the performance of the obligate, nutritional symbiont *Portiera aleyrodidarum*. Relative to aphid-associated strains, whitefly-associated *H. defensa* have modestly reduced genomes (MEAM1; 1.7 Mb, MED 1.8 Mb vs. 2.1 Mb 5AT), including the loss of some pathogenicity factors that may influence localization and phenotype. Despite the smaller size, *B. tabaci* strains have retained genes, such as those involved in the biosynthesis of amino acid biosynthesis that potentially complement losses in *P. aleyrodidarum* (Rao et al. 2015; Rollat-Farnier et al. 2015). However, metabolic modeling suggests that *H. defensa* may primarily be a nutritional parasite of its whitefly host and *Portiera* rather than a mutualist (Ankrah et al. 2017). Consistent with either role, *B. tabaci*-associated *H. defensa* appear restricted to bacteriocytes where they co-occur with *P. aleyrodidarum* (Gottlieb et al. 2008; Rao et al. 2015). In contrast, *H. defensa* primarily persists extracellularly in the hemocoel of aphids (Brandt et al. 2017).

Altogether, in vivo studies implicate *H. defensa* and APSEs in activities that affect host fitness. In contrast, insights into trait variation are limited because the genome of only one aphid-associated strain (*A. pisum* 5AT) has been fully assembled (Degnan et al. 2009), while only partial genome assemblies are available for *H. defensa* from *B. tabaci* MED and MEAM1. Obligate symbionts like *Buchnera* have highly reduced genomes with stable architectures when compared with free-living bacteria (Moran et al. 2008), while facultative symbionts, including *H. defensa* show only moderate genome reduction (Oliver et al. 2010). Generating fully assembled genomes for facultative symbionts is often hindered by the small size of host insects and low abundance of symbiont cells, which makes isolation of high quality sequencing templates difficult. Collection of *H. defensa* from aphid hemolymph and whitefly bacteriocytes coupled with whole genome amplification provided the templates used for sequencing the 5AT strain by Sanger and pyrosequencing methods, and the MED and MEAM1 strains by Illumina (Degnan et al. 2009; Rao et al. 2012; Rollat-Farnier et al. 2015). However, assemblies for MED and MEAM1 *H. defensa* lack resolution of mobile genetic elements (MGEs) and contain gene fragments split across contigs that cannot be assembled. Thus, the absence of sequence data from other aphid-associated strains limits insights the 5AT genome can provide about strain variation, while short-read genome sequencing of MED and MEAM1 cannot identify large-scale rearrangements of potential importance in strain variation.

To generate complete genome assemblies for additional strains, we took advantage of recently developed methods for in vitro culture of *H. defensa* (Brandt et al. 2017). This allowed us to collect symbiont DNA without amplification or contamination by aphid or *Buchnera* DNA. This material was then used with single molecule real-time (SMRT) sequencing (PacBio) to generate complete genomes for four *H. defensa* strains from *A. pisum* that vary in APSE infection and strength of protection against parasitoids. Strain A2C is uninfected by APSE and provides no protection against wasps; strain AS3 contains APSE3 and confers high levels of protection; and strains NY26 and ZA17 are infected by APSE2 and APSE8, respectively, and confer moderate levels of protection (Oliver et al. 2009; Martinez et al. 2014; Oliver and Martinez 2014; Doremus and Oliver 2017). We also generated RNAseq data that was mapped to the A2C, AS3, and ZA17 genomes. Our results overall show that MGEs strongly affect genome organization and content between strains, while also identifying base modification differences and transcriptional heterogeneity over genome-wide scales.

## Materials and Methods

### *Hamiltonella defensa* Strains

*A. pisum* lines of identical genetic background were previously established from single parthenogenetic females that hosted the phage-free A2C, APSE3-infected AS3, APSE2-infected NY26, and APSE8-infected ZA17 strains of *H. defensa* (Oliver et al. 2009, 2012; Martinez et al. 2014; Doremus and Oliver 2017). In vitro cultures were established from these aphid lines and maintained in 25 cm^2^ culture flasks (Falcon) under ambient atmosphere at 27° C in 5 ml of TC100 medium (Sigma) plus 10% of fetal bovine serum (FBS) (Hyclone) (Brandt et al. 2017). The NY26 strain performs poorly at 27° C (Brandt et al. 2017) and instead was maintained at 20° C in the same culture medium plus 1×10^4^ TN5 cells, which is an adherent cell line from the moth *Trichoplusia ni* (Brandt et al. 2017). A2C, AS3, and ZA17 were passaged weekly after growing to an average maximum density of 1×10^10^ bacteria per ml while NY26 was passaged biweekly after growing to the same density.

### Genome Sequencing, Assemblies, and Annotations

DNA was isolated from the A2C, AS3, and ZA17 strains after 240 passages in culture while DNA was isolated from NY26 strain after three passages. Each strain was grown in 8 ml of medium for 6 (A2C, AS3, ZA17) or 12 (NY26) days followed by pelleting at 6,000×*g* for 10 min. All NY26 grow extracellularly (Brandt et al. 2017). Any nonadherent TN5 cells in medium containing NY26 were removed by centrifugation at 50×*g*. The supernatant containing NY26 was then collected and centrifuged at 6,000×*g* for 10 min to pellet the bacteria. DNA from each strain was isolated using the DNeasy Blood and tissues kit (Qiagen) followed by titration using a NanoDrop (Thermo Scientific) and visualization of aliquots on a 1.5% agarose gel stained with ethidium bromide to verify DNA integrity. Samples were sent to the University of Massachusetts Medical School Core Facility for SMRTBell fragment library construction using Long-Insert Genomic DNA followed by SMRT sequencing. For each DNA strain, data were collected on a two SMRT Cell for 360 min with number of reads per strain ranging from 118,736 to 184,769 and read sizes ranging from 9,174 to 11,715 bp (supplementary table S1, Supplementary Material online).

De novo assemblies were performed with the Hierarchical Genome Assembly Process (HGAP.2) algorithm in the SMRT Portal (version 2.3.0) with standard parameters except for PreAssembler Filter v1 where minimum subread length was set to 16,000 bp to improve assembly at 30× coverage with only the longest reads. The origin of replication (OriRep) for each strain was identified by homology to 5AT and set at the end of the major contig (i.e., main bacterial chromosome). Contigs were circularized using Circlator (Hunt et al. 2015). Assembly coverage per strain was assessed by another run of the RS_resequencing.1 algorithm with the last version of contigs serving as references.

Coding sequence (CDS; =gene) predictions were performed using the NCBI Prokaryotic Genome Annotation Pipeline (PGAP) (Tatusova et al. 2016); accessed October 2016) and Rapid Annotation using the Subsystem Technology tool kit (RASTtk) (Brettin et al. 2015); accessed October 2016). Predictions from each annotation were merged and compared with *B*acterial G*E*nome *A*nnotation *C*omparis*ON* (BEACON) (Kalkatawi et al. 2015). Predictions for ribosomal (r), transfer (t), and transfer-messenger (tm) RNAs were kept from the PGAP annotation, while noncoding (nc) RNA predictions were performed with Infernal using the Rfam database version 12.1 (Nawrocki et al. 2015). Three classes of mobile genetic elements (MGEs) were annotated: transposable elements (TEs) present as insertion sequences (ISs), phage islands, and integrated plasmids. IS predictions were generated using the ISfinder website (Siguier et al. 2006); accessed October 2016) and ISsaga (Insertion Sequence semiautomatic genome annotation) tool (Varani et al. 2011) followed by manual curation. Phage island predictions were done with the web-based tool PHASTER (Arndt et al. 2016) and through manual annotation. For identification of larger and intact Myoviridae genomes we relied on Virfam (Lopes et al. 2014). Subsequent analysis of partial prophage fragments relied on alignment of homologous regions using Muscle (Edgar 2004) implemented in Geneious v.8.1 http://www.geneious.com, (Kearse et al. 2012) and NCBI BLAST (https://blast.ncbi.nlm.nih.gov/Blast.cgi; accessed January–February 2017). Plasmid islands were identified by BLASTn (Altschul et al. 1990). Identification and classification of secretion systems was performed with TXSScan (Abby et al. 2016). KEGG Orthology (KO) was assessed by the automatic annotation servers BLASTKOALA (KEGG Orthology And Links Annotation) (Kanehisa et al. 2016). Putative functions of DNA methyltransferases (MTases) and restriction endonucleases (REases) were assessed by BLASTP against the restriction enzyme database (REBASE) (http://rebase.neb.com/rebase/rebase.html). Annotation files corresponding to the above-described annotation pipeline for each strain plus the reannotation of the 5AT strain are available as supplementary table S1, Supplementary Material online.

### Phylogenetic Reconstruction

Degnan and Moran (2008b) generated an *H. defensa* phylogeny based on eight partial gene sequences (*accD*, *dnaA*, *gyrB*, *hrpA*, *murE*, *ptsI*, *recJ*, and *rpoS*) from multiple strains*.* Accession numbers for this data set were downloaded from NCBI Batch Entrez (https://www.ncbi.nlm.nih.gov/sites/batchentrez). Corresponding annotated sequences were gathered from A2C, AS3, NY26, ZA17, and 5AT. Sequences were also downloaded as fasta files and annotated using RAST-Classic (December 2016) (Aziz et al. 2008) from: the *H. defensa* MED strain (NCBI assembly ASM25834v2), *H. defensa* MEAM strain (NCBI assembly AQE_PRJEB7127_v1), *Regiella insecticola* LSR1 (EnsembleBacteria SM14362v1), *Yersinia pestis* (EnsembleBacteria YAU: DJ56), *Y. enterocolitica* (EnsembleBacteria YEA: DJ62), *Photorhabdus asymbiotica* (EnsembleBacteria ASM19647v1), *P. luminescens laumondii* (EnsembleBacteria ASM19615v1), and *Escherichia coli* K-12 V.87.1 (EnsembleBacteria assembly ASM80076v1). 5AT orthologs were identified using tBLASTx (NCBI BLAST 2.5.0+, build September 9, 2016 13:36:03), aligned using MUSCLE v.3.8.31 (Edgar 2004) and manually checked using Geneious (Kearse et al. 2012). Full-length genes were then used to identify orthologs in the other strains and outgroups using tBLASTn or tBLASTx (Altschul et al. 1990) followed by alignment by MUSCLE (Edgar 2004) implemented in Geneious (Kearse et al. 2012). Aligned orthologs were trimmed to the length of the partial gene sequences used by Degnan and Moran (2008b), and then concatenated into a single matrix (supermatrix) using Geneious (Kearse et al. 2012). PartitionFinder v.2.1.1 (running Python v.2.7.12: Anaconda 4.2.0; Lanfear et al. 2012) was used to select an optimal data partition (when considering all codon positions in each gene individually) and nucleotide substitution models (considering only those models available to RAxML) using AICc. The optimal model was found to be GTR + gamma. Phylogenetic relationships were inferred from all sequence data simultaneously using RAxML (HPC v.8.2.8; Stamatakis 2014; random seed = 12345; provided with optimal partitions). Support for the phylogenetic tree was calculated from the percent of nodes recovered in 100 bootstrap replicates completed using RAxML. The tree was viewed and a figure generated using FigTree v.1.4.3 (tree.bio.ed.ac.uk/software/figtree).

### Comparative Genomics

Whole genome alignments were performed with MAUVE (Darling et al. 2004, 2010), which allowed us to manually curate genome annotations for each strain using Geneious (Kearse et al. 2012). Manual curation focused on normalizing start codons for homologous genes in different strains and annotation of pseudogenes. Most pseudogenes identified by PGAP were kept with the exception of those with “missing start” or “missing stop” tags. We further categorized a given CDS as a pseudogene when an internal stop codon was identified that led to a size reduction <80% of the size of the same gene in other strains. Genes with an IS insertion that led to predictions of different CDSs were also manually annotated as pseudogenes. When a noncoding (nc) RNA was not predicted in a given strain because of single nucleotide polymorphisms (SNPs), we manually annotated the ncRNA as a pseudogene. Finally, we applied the same pipeline of annotation to the 5AT strain (NCBI Reference Sequence: NC_012751) to generate an updated version for this genome so that more accurate comparisons could be made between strains. Manually built “Pan-genomes” were subdivided into a “Core genome” containing genes present in all strains, an “Accessory genome” containing genes in two or more strains, and a “Unique genome” containing genes in only one strain (Tettelin et al. 2005; Chaudhari et al. 2016).

Protein coding sequences were extracted from the five sequenced strains of *H. defensa* from *A. pisum* and two sequenced strains from *B. tabaci* as nucleotide sequences followed by identification of orthologous groups using OrthoFinder (Emms and Kelly 2015). Gene sequences were then translated and aligned using PASTA (v.1.7.8) (Mirarab et al. 2015) under default settings. A Maximum likelihood gene tree was calculated for each orthologous group using FastTree (v.2.1.7; Price et al. 2010) from the amino acid alignments. The aligned amino acid sequences were then back translated to the gene sequences maintaining the alignment. Codeml (implemented in paml v.4.9; Yang 1997) was used to generate pairwise estimates of nonsynonymous substitutions per nonsynonymous site (dN) and synonymous substitutions per synonymous site (dS).

### Base Modification

Base modification analysis and motif detection were performed using the RS_Modification_and_Motif_Analysis.1 algorithm in the SMRT Portal with standard settings (minimum-modification QV of 30). To reduce false positive motif detection due to high level base coverage, we reanalyzed the data output with specific scripts provided in Base Modification Tools accessible on the Pacific Biosciences GitHub pages (https://github.com/PacificBiosciences/Bioinformatics-Training/wiki/BaseModification-Tools) using a minimum-modification QV of 100 for base modification and motif prediction. Motifs were then manually curated per PacBio guidelines. Methylome maps for each strain were made using ClicO and Circos (Krzywinski et al. 2009; Cheong et al. 2015).

### Transcriptome Sequencing and Mapping

Total RNA was isolated from the A2C, AS3, and ZA17 strains 5 days after inoculating into culture flasks using the mirVana miRNA Isolation Kit (Ambion, Thermo Fisher Scientific) followed by DNase treatment using the TURBO DNA-free Kit (Ambion, Thermo Fisher Scientific) and ethanol precipitation in the presence of glycogen. A total of two (A2C) or three (AS3, ZA17) separate cultures per strain were extracted generating two or three independent biological replicates. We sequentially depleted rRNA using the Ribo-Zero (Bacteria) Magnetic Kit (Epicentre, Illumina) according to manufacturer’s protocol followed by Ribo-Zero-Treated RNA purification using the Illumina modified RNeasy MinElut Cleanup Kit (Qiagen). RNA templates were quality checked by the Georgia Genomics Facility using an Agilent 2100 Bioanalyzer (Agilent Technologies) and Fragment Analyzer (Advanced Analytical). Standard, paired-end sequencing libraries (75 bp) were generated using the Kapa stranded RNA-seq Library Preparation Kit (Kapa Biosystems) and Illumina sequenced on a NextSeq (150 Cycles) Mid Output Flow Cell. RNAseq reads for each strain were aligned to their corresponding genomes using bowtie2 (Langmead and Salzberg 2012) with the stringent parameters –very-sensitive, –no-mixed, and –no-discordant. Read counts for each CDS were generated using htseq-count (Anders et al. 2015) while Reads per Kilobase of transcript per Million mapped reads (RPKM) were calculated as described by Mortazavi et al. (Mortazavi et al. 2008).

## Results

### Global Features of Aphid-associated *H. defensa* Genomes

The ability to culture *H. defensa* outside of *A. pisum* resulted in isolation of template DNA with no contamination from aphids, *Buchnera* or other bacteria*.* Our ability to isolate *H. defensa* from TN5 cells also resulted in almost no contamination from *T. ni*. SMRT sequence data for the A2C, AS3, NY26, and ZA17 strains were assembled and annotated, while the 5AT genome (Degnan et al. 2009) was reannotated for consistency using the same pipeline. Table 1 summarizes key annotation features while supplementary table S2, Supplementary Material online summarizes sequencing metrics. Each sequenced strain was assembled into a single contig for the main chromosome plus 3 (A2C, ZA17), 2 (AS3), or 0 (NY26) extrachromosomal plasmids (table 1). The reannotated 5AT genome consisted of a main chromosome and one extrachromosomal plasmid as previously reported (Degnan et al. 2009) (table 1). Strains had similar total genome sizes (∼2.2 Mb) and GC contents. Strains varied in terms of total CDSs (2,243 to 2,411) and ncRNAs (112 to 163) but were nearly identical in the number of transfer (t) RNAs (42 or 43) ribosomal (r) RNA operons (3) and transfer-messenger (tm) RNAs (1) they encoded (table 1). We could not circularize the main chromosome for any strain including 5AT because of palindromic regions at the ends of the contigs produced by de novo assembly (table 1). Only some extrachromosomal plasmids could be circularized for the same reasons. MGEs in the main chromosome of each strain consisted of TEs, prophage islands, and plasmid islands and coded for a total of 632 to 747 CDSs (table 1). Fifteen TE families were present in all strains while one (IS91) was restricted to NY26 and 5AT (supplementary fig. S1, Supplementary Material online). Copy number of each TE family member varied among strains with IS630 being most abundant in A2C and AS3, IS3 being most abundant in ZA17, and RtRdDp being most abundant in NY26 and 5AT (supplementary fig. S1, Supplementary Material online). Previous analysis of the 5AT genome identified 22 prophage and 11 plasmid islands (Degnan et al. 2009). Our comparative data set indicated that prophage islands ranged from 22 in 5AT and NY26 to 13 in A2C and AS3 while plasmid islands ranged 11 in 5AT and NY26 to 7 in A2C and ZA17 (table 1).

**Table 1:** Genome Features from Sequencing the *H. defensa* A2C, AS3, NY26, and ZA17 Strains and Reannotation of the 5AT Strain

	A2C					AS3				ZA17					NY26	5AT		
	Chromosome	pHDA2C.1	pHDA2C.2	pHDA2C.3	Total	Chromosome	pHDAS3.1	pHDAS3.2	Total	Chromosome	pHDZA17.1	pHDZA17.2	pHDZA17.3	Total	Chromosome	Chromosome	pHD5AT	Total
GC%	40.4%	46.2%	46.3%	46%		40.6%	46%	46.5%		40.5%	43.8%	47.9%	44.6%		40.3%	40.3%	45.3%	
Size (pb)	1,997,367	84,195	72,453	50,279	2,204,294	2,054,032	140,172	67,631	2,261,835	2,104,645	98,627	38,147	31,227	2,272,646	2,122,402	2,110,331	59,032	2,169,363
Circularization	no	yes	No	yes		No	yes	no		no	no	no	yes		no	no	yes	
Coding density (pseudo)	87.4% (8.3%)	89.8% (13.2%)	85.1% (12.7%)	89.5% (16.4%)	87.5% (8.8%)	87.7% (11.5%)	89.4% (12.1%)	85.0% (17.0%)	87.8% (11.7%)	87.4% (7.8%)	80.2% (18.9%)	89.7% (18.7%)	83.9% (13.2%)	87.1% (8.6%)	86.7% (17.2%)	86.9% (11.3%)	85.4% (13.4%)	86.8% (11.4%)
CDS (pseudo)	2,116 (156)	85 (7)	78 (8)	68 (7)	2,347 (178)	2,181 (200)	161 (16)	69 (12)	2,411 (227)	2,185 (170)	91 (16)	50 (7)	44 (5)	2,370 (198)	2,243 (282)	2,241 (203)	65 (7)	2,306 (214)
ncRNA (pseudo)	98 (13)	10	4	6	118 (13)	99 (13)	16	3 (1)	118 (14)	99 (11)	7 (2)	2	4	112 (13)	162 (18)	158 (18)	5	163 (18
rRNA	9	0	0	0	9	9	0	0	9	9	0	0	0	9	9	9	0	9
tmRNA	1	0	0	0	1	1	0	0	1	1	0	0	0	1	1	1	0	1
tRNA	43	0	0	0	43	43	0	0	43	42	0	0	0	42	42	42	0	42
Palindromic region	2	0	2	0	4	1	0	2	3	2	2	0	0	4	2	1	0	1
TEs CDS	183	12	10	6	211	184	18	10	212	124	14	4	6	148	198	195	1	196
Prophage island	13	0	0	0	13	13	0	0	13	21	0	0	0	21	22	22	0	22
Plasmid island	7	0	0	0	7	8	0	0	8	7	0	0	0	7	11	11	0	11
MGE (CDS)	632				863	683			913	682				867	747	745		810

Note.—Pseudo in parenthesis indicates the number of CDSs, or ncRNAs that are pseudogenized.

bp, base pairs; CDS, coding sequence; ncRNA, noncoding RNA; rRNA, ribosomal RNA; tRNA, transfer RNA; tmRNA, transfer-messenger RNA; TE, transposable element; MGE, mobile genetic element.

### Aphid-associated *H. defensa* Reside in Distinct Clades and Show Extensive Horizontal Transfer among Hosts

**Fig. 1.—:**
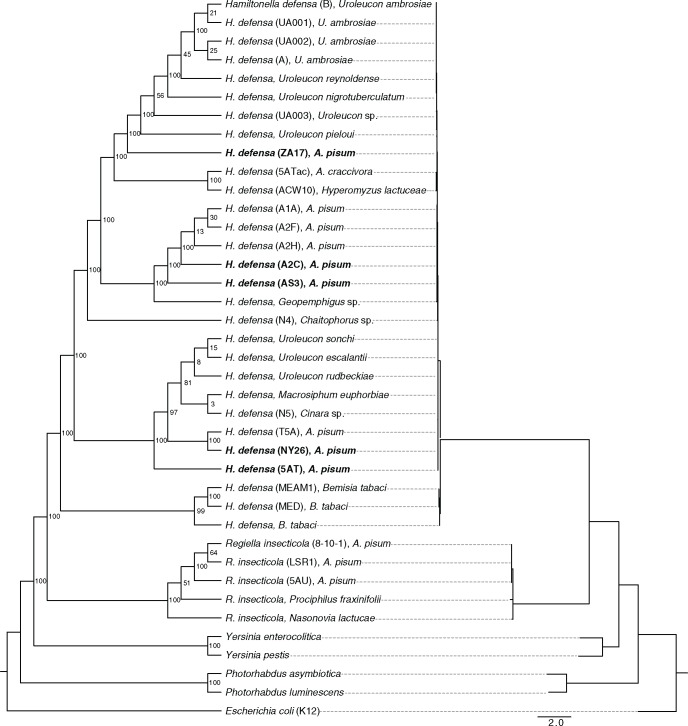
Maximum-likelihood cladogram (left) and phylogram (right) based on amino acid sequences of eight loci from 30 strains of *Hamiltonella defensa*, five strains of *Regiella insecticola* and selected outgroups. Taxon labels for *H. defensa* and *R. insecticola* indicate the name of the strain in parenthesis followed by the name of the aphid or whitefly host species. Taxon labels for outgroups indicate the species name and strain if appropriate. Bold font identifies the four strains of *H. defensa* from *Acyrthosiphon pisum* sequenced during the current study plus the reannotated 5AT strain. Numbers at nodes indicate support based on the percentage of 100 bootstrap replicates that recovered the same node. Scale bar for the phylogram indicates nucleotide substitutions per site.

We generated a maximum likelihood phylogeny to assess relatedness of the A2C, AS3, NY26, ZA17 strains to previously identified strains and outgroups (Degnan and Moran 2008b). Results were consistent with earlier conclusions (Degnan and Moran 2008b; Rollat-Farnier et al. 2015) that *H. defensa* is monophyletic and sister to the facultative symbiont *Regiella insecticola* (fig. 1). *H. defensa* in aphids and whiteflies formed two well supported clades as determined by bootstrap replicates, while aphid-associated strains subdivided into three clades albeit with a sizable split within the crown clade (fig. 1). Short branch lengths for the *H. defensa* clades indicated recent divergence. Strains with APSE2 phage (5AT and NY26) were found in the same clade, as were those associated with APSE3 (AS3, A2C, A1A, A2H, and A2F) (fig. 1). Note that strain A2C used in this study is APSE-free, but formerly carried APSE3, which was lost in laboratory held lines (Oliver et al. 2009). The phylogeny also indicated that each *H. defensa* clade contains representatives from different aphid taxa (often tribes) indicating extensive horizontal transmission. For example, the strains most related to 5AT/NY26 from *A. pisum* (Aphidinae: Macrosiphini) include one from distantly related *Cinara* (Lachinae: Eulachnini), and the closest relative to the APSE3-associated strains from *A. pisum* was sampled from *Geopemphigus* (Eriosomatinae: Fordini).

### Aphid-associated *H. defensa* Exhibit Rearrangements That Differ between Clades

**Fig. 2.—:**
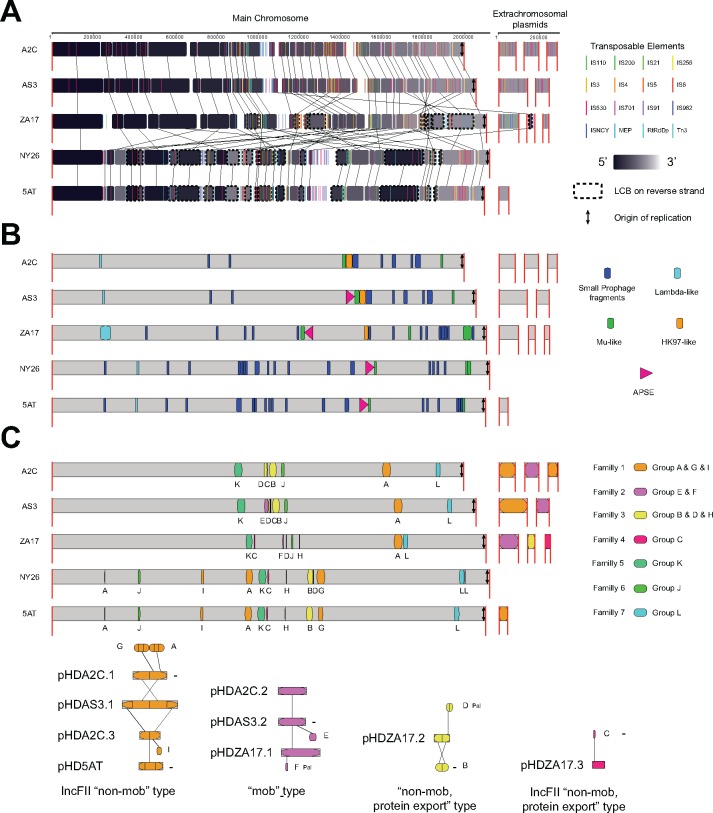
Whole genome alignments for *Acyrthosiphon pisum-*associated strains of *Hamiltonella defensa.* (*A*) The A2C strain was set as the reference with the main chromosome shown to the left and extrachromosomal plasmids shown to the right. Long red vertical bars indicate the boundaries of the main chromosome or extrachromosomal plasmids. Alignments are divided into Locally Collinear Blocks (LCBs) that correspond to regions of the genome in each strain that are internally free of rearrangements. Black blocks correspond to LCBs in the 5′ end and light gray blocks correspond to LCBs in the 3′ end of the main chromosome and extrachromosomal plasmids. Colored bars indicate the position of different TEs. Vertical arrows indicate the position of the origin of replication (*ori*) for each strain. Below A2C are alignments of the other strains. A dashed line surrounding an LCB indicates its reversal relative to A2C. (*B*) Location of prophage islands in the genomes of each strain. Dark blue indicates the location of small prophage fragments of different origins, while other colors indicate the location of APSE and other larger prophage islands present in all or particular strains*.* (*C*) Extrachromosomal plasmids and plasmid islands. The lower part of the figure illustrates the extrachromosomal plasmids. Plasmids that share homology are grouped by color and classification type. Internal vertical bars and letters identify domains in each plasmid while external vertical bars indicate homologous domains between plasmids. Plasmids in reverse complement are tagged with a minus sign “−”. The upper part of the figure shows the location of plasmid islands in the main chromosome. Islands derived from integration of extrachromosomal plasmids are indicated by the same color with letters corresponding to the domain of the extrachromosomal plasmid that still persists. Islands with other colors and letters derive from integration and decay of plasmids of unknown origin.

Using A2C as a reference, whole genome alignments indicated that genome organization was similar for strains in the same clade but differed between clades due to MGE-associated rearrangements (fig. 2*A*). Thus, the AS3 genome exhibited few differences relative to A2C outside of small deletions and the presence of APSE3 (fig. 2*A*). Comparison of A2C to ZA17 in contrast identified Locally Collinear Blocks (LCBs) that differed in position or were reversed, while comparison to 5AT and NY26 identified other rearrangements (fig. 2*A*). The majority of rearranged LCBs were flanked by the TEs that most differed in abundance between strains. For example, 6 of the 11 repositioned and/or reversed LCBs in ZA17 relative to A2C were flanked by IS3 while most repositioned and/or reversed LCBs in NY26 and 5AT were flanked by RtRdDp (fig. 2*A*).

Prophage islands were likewise similar in strains from the same clade but differed between strains in different clades (fig. 2*B*). Apart from APSE3, A2C, and AS3 contained small fragments from classifiable or unclassifiable phages plus a larger 24 kb HK97-like island (Myoviridae, Type 1, Cluster 3) (supplementary table S3, group B, Supplementary Material online). Each of these phage islands was also similarly positioned in the A2C and AS3 genomes (fig. 2*B*). The Hk97-like fragment contained several intact genes with functions in capsid formation, but other essential genes for replication were absent. ZA17 contained APSE8, a further reduced Hk97-like island, and several small phage genome fragments (fig. 2*B* and supplementary table S3, Supplementary Material online). It also contained two other large, potentially intact, prophages: a 46 kB lambda-like genome (Myoviridae Type 1 Cluster 6) (supplementary table S3, group D, Supplementary Material online) and a 38 kB Mu-like genome (Myoviridae Type 1 Cluster 8) (supplementary table S3, groups A and L, Supplementary Material online). Fragments of the lambda-like prophage were similarly positioned across strains. Small fragments of the Mu-like prophage resided elsewhere in the genome of ZA17 and other strains including one island that was always 3′ of each integrated APSE (fig. 2*B*). However, orientation of the APSE/Mu-like phage domain in ZA17 differed from the other strains because of repositioning and reversal of a flanking LCB (fig. 2*B*). 5AT and NY26 contained APSE2 plus several phage fragments related to those in other strains (supplementary table S2, Supplementary Material online). In contrast, the absence of an HK97-like island suggested this fragment had either been fully lost or an HK-97-like phage infected the common ancestor of ZA17, A2C, and AS3 after divergence from the 5AT-NY26 group (fig. 2*C*).

The extrachromosomal plasmid in the 5AT strain was previously named pHD5AT (Degnan et al. 2009). We similarly named the plasmids in the other strains pHDA2C.1, .2, and .3, pHDAS3.1 and .2, and pHDZA17.1, .2, and .3 (fig. 2*C*). Read mapping data were only 3- to 4-fold higher for each of these plasmids relative to the main chromosome, which suggested each was low copy. pHD5AT was partially homologous to pHDA2C.1, pHDA2C.3, and pHDAS3.1. In turn, pHDA2C.2 was partially homologous to pHDAS3.2 and pHDZA17.1, while pHDZA17.2 and pHDZA17.3 were distinct (fig. 2*C*). pHDA2C.1, pHDA2C.3, pHDAS3.1, and pHD5AT were classified as conjugative, nonmobilizable IncF11 plasmids on the basis that each encoded a RepA1 gene (Wu et al. 1992). pHDA2C.2, pHDAS3.2, and pHDZA17.1 encoded relaxase TraI-like genes associated with mobilizable type plasmids. pHDA2C.1 and pHDAS3.1 from the A2C and AS3 strains were further distinguished by encoding Toxin complex (Tc) and repeats in protein toxin (RTX)-like genes: two families that include proteins with insecticidal or lytic activities (Lally et al. 1999; Ffrench-Constant and Waterfield 2006; Hurst et al. 2011). pHDZA17.2 encoded virB4 and type 4 secretion system (T4SS) genes associated with nonmobilizable and protein export type plasmids, while pHDZA17.3 encoded RepA1, virB4, and T4SS genes associated with both conjugative and nonmobilizable protein export type plasmids. pHDZA17.3 also encoded a zinc metalloproteinase (Mpr) gene similar to plasmid pPAA3 identified from an Australian isolate of *Photorhabdus asymbiotica* (Wilkinson et al. 2010).

The 5AT main chromosome was previously reported to harbor two plasmid islands resulting from integration and decay of pHD5AT plus nine other plasmid islands of unknown origin (Degnan et al. 2009). Whole genome alignments revised this view by showing that a majority of the plasmid islands in *A. pisum-*associated *H. defensa* derive from extrachromosomal plasmids still present in some but not all of the strains we sequenced (fig. 2*C* and supplementary table S4, Supplementary Material online). However, some plasmid islands of unknown origin also still existed in each strain (fig. 2*C*). Like prophage blocks, the location of plasmid islands was similar in strains from the same clade but differed between clades due to LCB-associated rearrangements (fig. 2*C*).

### *H. defensa* Pan-Genome

**Fig. 3.—:**
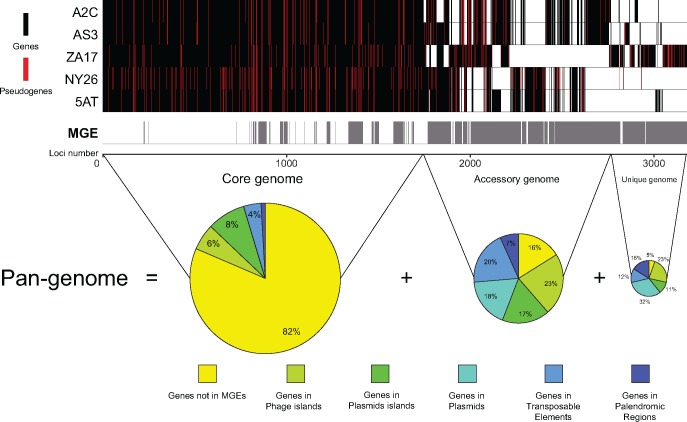
Genomic maps illustrating the Pan-genome for *Acyrthosiphon pisum-*associated strains of *Hamiltonella defensa*. Loci and Mobile Genetic Elements (MGE) in the Core, Accessory, and Unique genome are ordered along the *x* axis for each strain. Vertical bars symbolize orthologous genes. A black bar indicates the gene is present and putatively functional in a given strain, a red bar indicates the gene is present but pseudogenized, and no bar indicates the gene is absent. Gray bars below the strains indicate MGEs, which consist of phage islands, plasmid islands, plasmids, TEs, and palindromic regions. Note that most loci in the Accessary and Unique genome are associated with MGEs. Pie charts show the proportion of genes in the Core, Accessory, and Unique genome in different types of MGEs versus other domains.

SMRT sequencing generated an accurate, synteny-based pan-genome for *A. pisum*-associated *H. defensa* that consisted of 3,179 loci subdivided into: 1) a core genome of 1,745 loci in all strains, 2) an accessory genome of 1,019 loci in some but not all strains, and 3) a unique genome of 415 loci present in only one strain (fig. 3). Most core genes (82%) had housekeeping functions (metabolism, transport, cell envelope) while the balance were associated with MGEs present in all strains (18%). As expected, most accessory (84%) and unique genes (95%) were associated with MGEs restricted to a subset of strains or a particular strain (fig. 3). A total of 518 loci in the pan-genome were pseudogenized with the number of pseudogenes per strain ranging from 177 in A2C to 282 in NY26 (table 1). Most pseudogenes were due to point mutations that introduced premature stop codons or frameshifts, although some arose from MGE insertions that subdivided CDSs (supplementary fig. S2, Supplementary Material online). Most pseudogenes produced by MGEs were associated with the IS630 TE in the A2C-AS3 group or the RtRdDp TE in the 5AT-NY26 group. Only 42 loci in the core genome were pseudogenized in all five strains of which only 13 were not in MGEs (supplementary fig. S2, Supplementary Material online). These included four genes in an operon coding for a glycosyltransferase and a GHMP kinase, a phosphomannomutase, three RTX toxins, a Fic/DOC protein family member, a DNA binding protein, a transcriptional regulator, and two hypotheticals. Most pseudogenes in the accessory genome were shared between the A2C, AS3, and ZA17 or were shared between 5AT, NY26, and ZA17. Most other pseudogenes co-occurred in strains from the same clade or were strain specific, which suggested inactivation occurred more recently (supplementary fig. S2, Supplementary Material online).

**Fig. 4.—:**
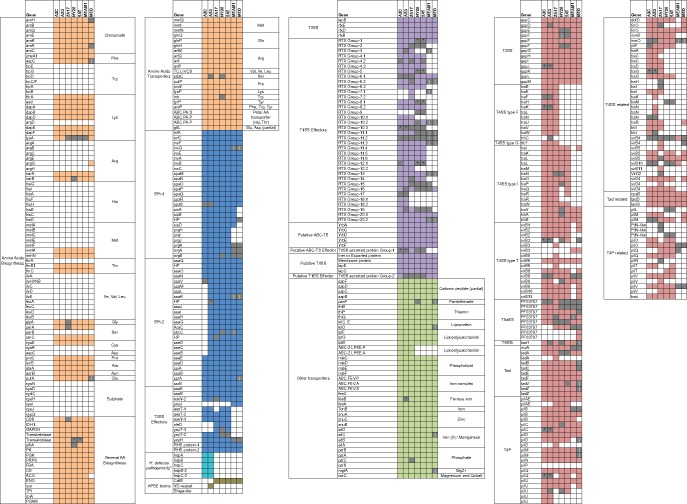
Gene content associated with specific functional activities in *Acyrthosiphon pisum-* and *Bemisia tabaci-*associated *Hamiltonella defensa*. These include: amino acid biosynthesis* and transport (brown boxes), Type 3 secretory systems (T3SS SPI-1, SPI-2) and their effectors (blue boxes), the *H. defensa* pathogenicity** cluster (aqua boxes), APSE toxins (olive boxes), Type 1 secretory system (T1SS) and effectors in the RTX toxin family, a putative group 2 T1SS plus effectors (purple boxes), other transporters with predicted functions in acquisition of growth factor including vitamins (light green boxes), and Type 2 and Type 4 secretory systems (T2SS, T4SS), Tight adherence (Tad) transport system, and Type VI pilus (T4P) components (red boxes)***. For each strain, a colored box indicates the gene is intact and putatively functional, an open box indicates the gene is absent, and a gray box indicates the gene is present but pseudogenized. Pseudogenization by point mutation is indicated by a black dot while pseudogenization by MGE-associated breakage is indicated by a vertical line. * Open boxes present in all strains regarding genes from amino acids biosynthesis correspond to genes present in *Buchnera aphidicola* (adapted from Rao et al. 2015). ** *H. defensa* pathogenicity refers to the gene cluster on pHDA2C.1 and pHDAS3.1 with similarity to the *Serratia entomophila* lysis cluster on the plasmid pADAP. *** Genes present in T4SS-, Tad-, and T4P-related boxes correspond to genes with similarities with T4SS, Tad, and T4P identified by our annotation pipeline but were not identified by the specific secretion system prediction tool TXSScan (Abby et al. 2016).

The pan-genome for aphid-associated *H. defensa* together with the draft genomes for whitefly-associated *H. defensa* (MED, MEAM1) suggested all strains are aerobic heterotrophs with intact glycolytic, tricarboxylic acid (TCA), and pentose phosphate pathways, but are host dependent because of similar losses of genes required for amino acid biosynthesis (fig. 4). Only a few losses of genes with functions in amino acid biosynthesis differed between strains. These included *argG*, which is absent from all strains except MED, an aconitate hydratase (*ACO*) absent in MED but present in all other strains, and *lysA* that is pseudogenized in four strains (ZA17, NY26, 5AT, and MEAM1) but is intact in three others (A2C, AS3, and MED) (fig. 4). In contrast, substrate-specific transporters are largely conserved across strains (fig. 4). These data in combination with the ability to culture multiple strains in vitro (Brandt et al. 2017) indicates most if not all *H. defensa* associated with aphids acquire missing nutrients from their environment.

In contrast, the inventory of loci with putative roles in pathogenicity was more variable. The two type III secretion systems (T3SS) in 5AT (SPI-1 and -2) (Degnan et al. 2009) were previously reported to be defective in MEAM1 and MED due to loss of most T3SS effectors and several SPI-1 genes (Rollat-Farnier et al. 2015). Our results show variable losses or pseudogenization in T3SS effectors across strains with only A2C showing severe defects in SPI-2 due to a 15-kb deletion that eliminated 14 genes (fig. 4). Other virulence factors in *H. defensa* include a type I secretion system (T1SS) and multiple RTX family members (Degnan et al. 2009; Rollat-Farnier et al. 2015). MEAM1 and MED were previously reported to encode most T1SS genes identified in 5AT but a reduced number of RTX genes (Rao et al. 2015; Rollat-Farnier et al. 2015). Our results indicate the T1SS secretion system (Group 2) is conserved across strains with variable defects in RTX genes that include extensive losses of Group 7 and 18 family members and pseudogenized Group 2 family members in AS3, ZA17, NY26, and 5AT (fig. 4). We also identified a type II secretion system (T2SS), which appears to be intact in ZA17, MED, and MEAM1, whereas some components are pseudogenized in A2C, AS3, NY26, and 5AT (fig. 4). Several type IV secretion system (T4SS) components were also identified with some strain specificity (fig. 4). For example, T4SS type F was specific to A2C, AS3, and ZA17, while T4SS type I was specific to *H. defensa* from *A. pisum*. T4SS type T was shared by all strains but was only fully intact in ZA17 while exhibiting strain-specific defects in A2C, AS3, and NY26 (fig. 4). We also identified several components of the Tight adherence (Tad) macromolecular transport system in all strains but with some defects in NY26, 5AT, MED, and MEAM1.

Multiple copies of different components of the Type VI pilus (T4P) were identified in all strains (fig. 4). We also identified conserved and active phospholipid transporter cassettes with functions in outer membrane vesicle (OMV) assembly (Roier et al. 2016) that are clustered with a second T1SS secretion system (Group 1) that putatively secretes an Ig-like domain repeat protein (fig. 4) recently implicated in pathogenicity of *Haemophilus influenzae* (Roier et al. 2016). However, ZA17 is the only strain in which this T1SS is potentially active (fig. 4). As previously noted, plasmid-encoded TC-like toxins were restricted to A2C and AS3 (fig. 4). These genes were most similar in organization to the TC-like toxins designated as the Sep lysis cassette in the extrachromosomal plasmid pADAP from *Serratia entomophila*: an insect pathogen that causes amber disease (Hurst et al. 2011). We therefore named these elements the *H. defensa* pathogenicity (hdp) A, B, C, B-2, C-2 genes (fig. 4). For APSEs, previously described toxin cassettes included a YD-repeat gene specific to APSE3 that infects AS3, and very similar but not fully identical variants of a cytolethal distending toxin (*cdt*) gene present in APSE2 and APSE8 that infect 5AT, NY26, and ZA17, and the APSEs that infect MED and MEAM1 (Degnan and Moran 2008a; Rao et al. 2015) (fig. 4).

We analyzed nonsynonymous (dN) and synonymous (dS) substitution frequencies per site to quantify sequence divergences across positional orthologs shared by all strains of aphid- and whitefly-associated *H. defensa*. Mean dN (0.001–0.03) and dS (0.002–0.111) were overall very low (supplementary table S5, Supplementary Material online) with the lowest values being between aphid strains in the same clade and the highest values being between aphid and whitefly strains (supplementary table S4, Supplementary Material online). No genes exhibited dS values >0.6 in comparisons between A2C, ZA17, and NY26 while a small number of genes exhibited dS values >0.6 in comparisons between aphid and whitefly associated strains.

### Structure of the *H. defensa* Global Transcriptome

**Fig. 5.—:**
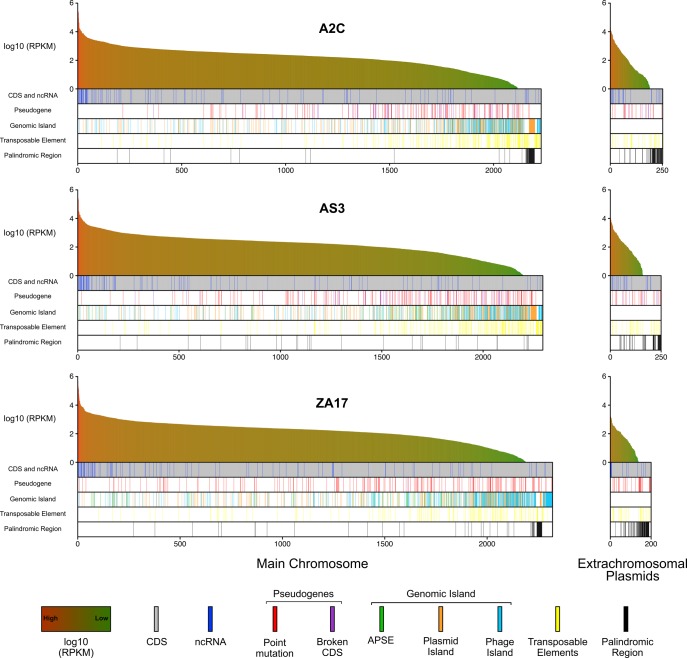
Transcriptional profiling by RNAseq of in vitro cultured A2C, AS3, and ZA17. For each strain, RPKM values (log10) are ordered from highest (red) to lowest (green) for all CDSs (gray bars) and ncRNAs (blue bars) in the main chromosome (left) and extrachromosomal plasmids (right). CDSs that have been pseudogenized by point mutation or MGE insertion (Broken CDS) are indicated. CDSs and ncRNAs in APSEs, other phage and plasmid islands, transposable elements, or palindromic regions are also indicated.

RNAseq data provide an unbiased approach for assessing genome annotations, but global transcriptome analyses have not previously been reported for any heritable insect symbiont because of difficulties associated with mRNA enrichment when bacterial abundance is limited. However, our ability to culture multiple strains of *H. defensa* overcomes this constraint, which prompted us to generate global expression profiles. The total number of reads generated per sample ranged from 20.3 to 46.9 million pairs with >97% for each sample mapping to whole genome assemblies (supplementary table S6, Supplementary Material online). Mapped reads generated continuous distributions to genome assemblies for the main chromosome and extrachromosomal plasmids for each strain (fig. 5). Transcript abundances, as measured by RPKM, were highest for select chaperon proteins (*CspA, CspC, GroES, GroEL), DNAK*, and ribosomal proteins on the main chromosome (fig. 5). RPKM values for most predicted ncRNAs and APSE genes were high while genes in prophage islands and TEs were variable with some exhibiting high RPKM values and others exhibiting low or zero values. Overall, the lowest RPKM values were associated with pseudogenes and genes in prophage and plasmid islands (fig. 5). Variability in reads mapping to palindromic regions could potentially reflect the restrictive parameters used since palindromes by nature could induce discordant reads.

### Restriction Modification System

Bacteria and other prokaryotes ubiquitously encode DNA methyltransferases (MTases), which together with restriction endonucleases (REases) form restriction modification (RM) systems that provide defense against bacteriophages and other sources of foreign DNA (Vasu and Nagaraja 2013). Bacteria also commonly encode orphan REases and MTases with other potential functions, including epigenetic regulation, while also showing evidence of horizontal acquisition via plasmids, phages, and other MGEs (Murphy et al. 2013). Genome-wide base modification analysis generated by SMRT sequencing identified 875 to 17,139 ^m6^A modifications, 24,457 to 31,542 ^m4^C modifications, and 75,352 to 83,428 unidentified modified bases across the A2C, AS3, NY26, and ZA17 genomes (supplementary fig. S3, Supplementary Material online). Motif prediction identified three putative MTase recognition motifs: G^m6^ATC, GC^m6^AN_6_TCC, and CG^m6^AN_6_TCC. Notably, these recognition motifs varied between strains with the G^m6^ATC motif most commonly modified in A2C and NY26, GC^m6^AN_6_TCC most commonly modified in ZA17, and CG^m6^AN_6_TCC specifically modified in NY26 (supplementary fig. S3 and table S7, Supplementary Material online).

Annotation identified a total of nine loci with potential roles in base modification, epigenetic regulation, and/or RM. Notably, five of these loci resided in prophage Islands while one resided in a plasmid island (supplementary table S8, Supplementary Material online). ZA17 possessed all nine loci including locus 1, 7, and 8 that harbor a type II MTase and a type II REase. Locus 1 is pseudogenized by point mutation in A2C and AS3, locus 7 is conserved in NY26 and 5AT but the MTase is pseudogenized in both. Only five RM loci were identified in A2C and AS3 (supplementary table S6, Supplementary Material online). Locus 8 was a strong candidate for modifying the motif GC^m6^AN_6_TCC in ZA17 and CG^m6^AN_6_TCC in NY26, because the MTase and REase appear to be functional in both strains and a divergent region in the gene coding for the specificity enzymes could underlie the difference in recognized motifs between strains (supplementary table S8, Supplementary Material online). Locus 2 in contrast is a strong candidate for modifying the G^m6^ATC motifs in A2C and NY26, because this locus codes for an orphan DNA adenine methyltransferase (Dam) identified as modifying the GATC motif in REBASE. Moreover, in AS3 and ZA17, which show no modification at the GATC motif, this orphan MTase is pseudogenized by point mutation (supplementary table S8, Supplementary Material online).

## Discussion

Strain variation has been reported for several heritable facultative symbionts including *H. defensa* (Perlman et al. 2006; Werren et al. 2008; Hansen et al. 2012; Hamilton and Perlman 2013; Oliver and Martinez 2014). However, little or no comparative genomic information based on complete genome assemblies is available for most species, which limits understanding of how strains differ and the suite of traits that potentially affect the function of facultative symbionts in their interactions with hosts. In the case of insects, this deficiency is primarily due to the low abundance of many facultative symbionts and small size of many host species. In this study, we show that recently developed methods for culturing *H. defensa* outside of *A. pisum* together with SMRT sequencing produced complete genome assemblies for four strains of *H. defensa* that differ in the protection levels they confer against parasitoids. Phylogenetic results further indicate these strains fall into three clades, which together with data generated previously for 5AT, MED, and MEAM1 provide new insights on strain variation.

Our results indicate that aphid-associated strains of *H. defensa* share most genes with roles in nutrient acquisition, metabolism, and essential housekeeping functions. These similarities also extend to the MED and MEAM1 biotypes of *B. tabaci.* In contrast, the aphid-associated strains we sequenced plus 5AT differ in regard to the total inventory of MGEs they harbor, which results in strain-specific differences in gene content and genome architecture. The diversity of extrachromosomal plasmids and plasmid islands in *H. defensa* is much greater than previously recognized, while the association of ISs with rearranged LCBs suggests a key role for mobile elements in shaping genome architecture through recombination. A substantial number of phage and plasmid-associated genes are also strain specific. These include previously unknown toxin genes that expand the inventory of potential virulence factors beyond previously reported factors encoded by APSEs (Moran et al. 2005; Degnan and Moran 2008a; Oliver et al. 2012; Dykstra et al. 2014; Martinez et al. 2014; Dennis et al. 2017). The presence of largely intact lambda and Mu prophages in ZA17 and Tc-like toxin genes in A2C and AS3 plasmids suggests these elements have been acquired since divergence from the other strains we sequenced. Overall, strains assigned to the same clade share greater similarity in genome synteny, gene content, and MGE inventory than strains in different clades. This finding also supports the use of multilocus sequence typing approaches for phylogenetic assignment of *H. defensa* strains (Degnan and Moran 2008b; Ferrari et al. 2012; Henry et al. 2013; Russell et al. 2013; Brandt et al. 2017).

Within clade comparisons link some MGE-associated features to protection against parasitoids. For example, A2C and AS3 differ greatly in protective phenotype with the former providing no defense against parasitoids and the latter providing high defense (Martinez et al. 2014). Gene content and synteny are overall very similar between these strains with only modest differences in pseudogenization of particular genes and gene content of extrachromosomal plasmids and plasmid islands. However, A2C substantially differs from AS3 in regard to a 15 kb deletion that eliminates most SPI-2 T3SS genes while also lacking an APSE, which suggests one or both of these underlie the strong protective phenotype exhibited by AS3. Recent functional experiments narrow the protective phenotype of AS3 against the parasitoid *Aphidius ervi* to APSE3 by showing that transfer of this virus to A2C confers a fully protective phenotype despite the absence of SPI-2 genes (Brandt et al. 2017). A2C and AS3 are also unique among the strains we sequenced in that both harbor extrachromosomal plasmids encoding Tc genes most similar to the Tc genes of *Serratia entomophila* that causes amber disease in certain Coleoptera (Hurst et al. 2011). The absence of a protective phenotype in A2C without APSE3 transfer strongly suggests Tc genes do not have a direct role in defense against *A. ervi.* However, these toxin genes may have functions against other natural enemies.

Strains from different clades (e.g., A2C/AS3 vs. 5AT/NY26 or ZA17) also differ in the protective phenotypes they confer (Martinez et al. 2014), but associating these differences with particular traits is much more difficult due to greater variation in gene content. As previously noted, 5AT/NY26 and ZA17 provide moderate protection against *A. ervi* (Oliver et al. 2005, 2009; Doremus and Oliver 2017; Martinez AJ et al. 2017) in *A. pisum*, and are each infected by similar APSEs (2 and 8) that encode CdtB toxin genes. Yet unlike APSE3-infected AS3, in vitro experiments indicate that APSE8-infected ZA17 does not directly kill developing *A. ervi* (Brandt et al. 2017), which suggests both *H. defensa* and aphid-produced factors are required for the protective phenotype observed in vivo. We also note that secretion systems and associated effector genes previously hypothesized to mediate entry of *H. defensa* into host cells (Degnan et al. 2009) or toxin delivery to potential competitors (Green and Mecsas 2016) exhibit variable patterns of loss or pseudogenization across strains, which make it unclear whether any have essential roles in protection against parasitoids.

Incomplete assemblies for other facultative symbionts including *Regiella insecticola* and *Serratia symbiotica* preclude full assessment of large-scale genome rearrangements but do implicate MGEs in strain differentiation (Degnan et al. 2010; Burke and Moran 2011; Manzano-Marín et al. 2012) as documented in this study. The role of mobile elements in bacteria that have recently transitioned to insect symbiosis is also known (McCutcheon and Moran 2012). Heritable symbionts with broad host ranges, such as *Wolbachia pipientis*, share features with *H. defensa* that include an abundance of TEs with likely roles in genome rearrangements and horizontal gene transfer events (Bordenstein and Wernegreen 2004; Baldo et al. 2006; Klasson et al. 2009). Variation among *Wolbachia* strains has also been implicated in variable effects on hosts that include differential protection against pathogens (Martinez J et al. 2017) and a key role for phage genes in regulating cytoplasmic incompatibility (LePage et al. 2017). The co-occurrence of multiple *H. defensa* strains in some populations of *A. pisum* and the capacity for horizontal transfer (Oliver et al. 2010; Russell et al. 2013; Łukasik et al. 2015) provides opportunity for recombination. However, unlike *Wolbachia* (Baldo et al. 2006; Klasson et al. 2009) our results indicate little strain variation exists among core genes, which overall suggests a stable genomic background for *H. defensa* in association with high fidelity vertical transmission.

Although obligate symbionts like *B. aphidicola* exhibit highly stable genome architectures (McCutcheon and Moran 2012; Land et al. 2015), our results indicate that MGE-associated acquisition of novel genetic material and genome reorganization since host invasion is the key driver of strain diversity in *H. defensa*. Previous work by Russell et al. (2013) also supports this conclusion, but results of this study provide a more complete picture through use of new culturing and sequencing methods, and strategically sampling across the *H. defensa* phylogeny. Results from the current study indicate that MGE-mediated acquisitions and losses by facultative symbionts also share similarities with many free-living bacteria (Kumwenda et al. 2014; Raeside et al. 2014; Land et al. 2015; Argemi et al. 2017). Previous comparisons of *H. defensa* 5AT to its closest known relative, *R. insecticola*, indicate they share ∼55% of their genes with overall high dS values (>1.0) that are consistent with divergence >60 Ma (Degnan et al. 2010). The genomes of both species contain an abundance of MGEs, but exhibit very limited overlap in MGE-associated genes and a very low proportion of primarily plasmid-associated genes that show evidence of recent exchange (Degnan et al. 2010). Thus, while *H. defensa* and *R. insecticola* often coinfect aphids (Russell et al. 2013), unknown incompatibilities limit interspecific recombination and horizontal gene transfer.

In vitro culture enabled us to optimize conditions for generating RNA-Seq data from multiple *H. defensa* strains. Detailed analysis of this data set across *H. defensa* strains falls outside the focus of this study. However, we used these data to provide an independent assessment of our annotations while also generating absolute transcript abundances on a genome-wide basis. Expression levels of genes across strains of *H. defensa* exhibit a continuous distribution with no obvious divisions into discrete classes of CDSs expressed at high or low levels. This pattern is similar to mRNA expression levels reported for *Escherichia coli* and select other species of free-living bacteria, which is attributed to variation at the mRNA level among cells in a growing population (Passalacqua et al. 2009). In addition, notable is overall high transcript abundance levels for a number of ncRNAs, which include regulatory sRNAs that are known to affect transcription, translation, and stability of bacterial mRNAs through base pairing with targets (Barquist and Vogel 2015). RPKM values for several genes in phage islands are comparable with RPKM values for many *H. defensa* core genes. This suggests a number of phage genes besides toxins potentially contribute to *H. defensa* fitness as has been suggested in studies of select other bacteria (Bobay et al. 2014).

Our findings identify two potentially active RM systems in ZA17 and NY26 that recognize the motifs GCAN_6_TCC and CGAN_6_TCC. However, these motifs have not previously been characterized and are absent in REBASE, and will thus need further investigation. Our results also indicate the palindromic motif GATC is modified in A2C and NY26 but not AS3 and ZA17. Mechanistically, we hypothesize this difference is likely due to the DAM methylase gene being pseudogenized by point mutation in AS3 and ZA17. However, several functional questions remain regarding strains like A2C and AS3, which exhibit totally different methylation patterns for the GATC motif, yet have very similar genomes outside of infection by APSE3. Studies of other bacteria indicate that GATC methylation affects DNA repair, DNA replication, gene expression, and pathogenesis (Casadesus 2016). Recent experiments in *E. coli* further show that loss of adenine methylation at GATC sites results in growth defects (Westphal et al. 2016) and increased mortality during antibiotic stress (Cohen et al. 2016).

Our results further indicate that *H. defensa* is at continuous risk of infection by a variety of phages despite its long-term specialized association with insect hosts as a heritable facultative symbiont. SMRT sequencing further identifies strikingly different DNA methylation patterns as well as strain-specific differences in putative RM systems and orphan MTases in phage islands. The functional consequences of these findings are currently unclear but they suggest the possibility *H. defensa* strains differ in their susceptibility to invasion by different phages or other foreign DNA. A number of orphan MTases have also been identified in phages, which have been suggested to play roles in counter defense against host RM systems or as domesticated elements that enhance host fitness by affecting cell growth, DNA repair or other functions (Murphy et al. 2013).

In summary, our results provide the first comparative genomics data set on strain variation in a heritable facultative symbiont*.* Our results indicate that large-scale chromosomal rearrangements together with acquisition and losses of MGEs on short time scales substantially affect genome architecture of *H. defensa*. Given the phenotypic differences exhibited by these strains in defense against parasitoids, we further hypothesize genome rearrangements together with novel genes associated with particular MGEs affect strain fitness and interactions with host aphids.

## Supplementary Material

Supplementary data are available at *Genome Biology and Evolution* online.

## Supplementary Material

Supplementary Data
