# Supplementary material for: Culture-Facilitated Comparative Genomics of the Facultative Symbiont Hamiltonella defensa
Source: Genome Biol Evol. 2018 Feb 14;10(3):786–802. doi: 10.1093/gbe/evy036 (PMC5841374; doi:10.1093/gbe/evy036)
Supplement: Supplementary Data [file evy036_supp.zip › Supp_fig_legends.docx]

**Fig. S1.** Transposable elements (TEs) in *A. pisum-*associated strains of *H. defensa.* The number in parenthesis below the name of each strain indicates the total number of TEs present in the genome. Pie charts indicate the proportional abundance of each TE with percentages shown for the 4 most abundant TE types per strain. IS: Insertion Sequence; ISNCY: Insertion Sequence Not Characterized Yet; MEP: Mobile Element Protein; RtRdDp: Retron-type RNA-dependent DNA polymerase; Tn: Transposon.

**Fig. S2.**  Pseudogenes in the Pan-genome of *A. pisum-*associated *H. defensa*. Pseudogenized loci and Mobile Genetic Elements (MGE) in the Core, Accessary, and Unique genome are ordered along the x-axis. Vertical bars symbolize genes. A red bar indicates the gene is pseudogenized by point mutation, a purple bar indicates the gene is pseudogenized by MGE insertion, a black bar indicates the gene is intact, and no bar indicates the gene is absent. Gray bars below the strains indicate MGEs. Note that most loci in the core genome that are pseudogenized in all strains reside in MGEs.

**Fig. S3.** Methylome maps of *A. pisum-*associated *H. defensa* presented in Circos plots that show the main chromosome and extrachromosomal plasmids for each strain. The outermost ring shows the genomic island annotation: APSE (green), plasmid island (orange) and phage island (light blue). The next ring shows the CDS annotation for the positive and negative strand. Subsequent rings show specific base modification motifs: G**^m6^A**TC (blue), GC**^m6^A**N_6_TCC (green) and CG**^m6^A**N_6_TCC (purple). Motifs that are not associated with modified bases are shown in grey. The center of each Circos plot reports the total number of methylated cytosines (^m4^C) or adenines (^m6^A), modified bases, which refers to any potential modification that was not specifically called out by analysis software, but passed statistical thresholds for flagging and further investigation, and below threshold bases.
