# Supplementary material for: Culture-Facilitated Comparative Genomics of the Facultative Symbiont Hamiltonella defensa
Source: Genome Biol Evol. 2018 Feb 14;10(3):786–802. doi: 10.1093/gbe/evy036 (PMC5841374; doi:10.1093/gbe/evy036)
Supplement: Supplementary Data [file evy036_supp.zip › Table-S2.docx]

**Table S2**

Whole-genome Sequencing Metrics for the A2C, AS3, ZA17 and NY26 Strains of *H. defensa.*

|  |  |  |  |  |
| --- | --- | --- | --- | --- |
|  | A2C | AS3 | ZA17 | NY26 |
| Number of Bases | 1,695,148,175 | 1,391,049,249 | 1,525,871,564 | 1,654,041,412 |
| Number of Reads | 184,769 | 118,736 | 130,462 | 142,441 |
| N50 Read Length | 13,337 | 1,716 | 17,126 | 1,687 |
| Mean Read Length | 9,174 | 11,715 | 11,695 | 11,612 |
| Mapped Reads | 173,339 | 114,734 | 123,818 | 133,129 |
| % of Mapped Reads | 93,8% | 96,6% | 94,9% | 93,5% |
| Mean Coverage | 608 | 535 | 567 | 643 |
|  |  |  |  |  |
