# Supplementary material for: Culture-Facilitated Comparative Genomics of the Facultative Symbiont Hamiltonella defensa
Source: Genome Biol Evol. 2018 Feb 14;10(3):786–802. doi: 10.1093/gbe/evy036 (PMC5841374; doi:10.1093/gbe/evy036)
Supplement: Supplementary Data [file evy036_supp.zip › Table-S3.docx]

**Table S3**

Classification and Gene Inventory for the Phage Islands in the A2C, AS3, NY26 and ZA17 Strains of *H. defensa.* Phage Islands for 5AT are Summarized by Degnan et al. (2009).

| **Strain** | **Group** | **Classification** | **Lenght** | **Start** | **Stop** | **Structural Marker Gene inventroy** |
| --- | --- | --- | --- | --- | --- | --- |
| A2C | Group A | Partial Myoviridae of Type 1 Cluster 8, Mu-like | 21489 | 1414177 | 1435666 | Terminase large, portal |
| AS3 | Group A | Partial Myoviridae of Type 1 Cluster 8, Mu-like | 21489 | 1469976 | 1491465 | Terminase large, portal |
| NY26 | Group A | Partial Myoviridae of Type 1 Cluster 8 Mu-like | 10273 | 1554717 | 1564990 | portal, terminase large |
| ZA17 | Group A | Myoviridae of Type 1 Cluster 8, Mu-like, CHIMERIC | 36804 | 1996746 | 2033550 | Terminase small, terminase large, portal, major capsid, baseplate, baseplate assembly, tail fiber, tail fiber assembly, sheath, tail tube |
| ZA17 | Group A | Partial Myoviridae of Type 1 Cluster 8, Mu-like | 17089 | 1209051 | 1226140 | Terminase large, portal |
| A2C | Group B | Myoviridae of Type 1 Cluster 3, HK97-like | 23672 | 1435653 | 1459325 | Terminase small, terminase large, portal, major capsid, sheath, tail tube, tape measure, baseplate, baseplate assembly, tail fiber |
| AS3 | Group B | Myoviridae of Type 1 Cluster 3, HK97-like | 23672 | 1491452 | 1515124 | Terminase small, terminase large, portal, major capsid, sheath, tail tube, tape measure, baseplate, baseplate assembly, tail fiber |
| ZA17 | Group B | Myoviridae of Type 1 Cluster 3, HK97-like | 19496 | 1516942 | 1536438 | Terminase small, terminase large, portal, major capsid, sheath, tail tube, tape measure, baseplate, baseplate assembly, tail fiber |
| A2C | Group C | Nothing useful for classificaiton | 26741 | 1460826 | 1487567 | No marker genes present |
| AS3 | Group C | Nothing useful for classificaiton | 26739 | 1516625 | 1543364 | Terminase small |
| NY26 | Group C | Nothing useful for classificaiton | 16936 | 980774 | 997710 | Terminase small |
| ZA17 | Group C | Nothing useful for classificaiton | 8513 | 1536435 | 1544948 | No structural component genes present |
| A2C | Group D | Partial Myoviridae of Type 1 Cluster 6, lambda-like | 2887 | 233418 | 236305 | No structural component genes present |
| AS3 | Group D | Partial Myoviridae of Type 1 Cluster 6, lambda-like | 2886 | 248744 | 251630 | No structural component genes present |
| NY26 | Group D | Partial Myoviridae of Type 1 Cluster 6, lambda-like | 2643 | 413022 | 415665 | No structural component genes present |
| ZA17 | Group D | Myoviridae of Type 1 Cluster 6, lambda-like | 45585 | 246651 | 292236 | Terminase large, portal , major capsid, sheath, tail tube, tapemeasure, baseplate, tail fiber, tail fiber assembly |
| NY26 | Group E | Unclassified | 11731 | 909140 | 920871 | Sheath, tail tube, tape measure, baseplate, tail fiber, tail fiber assembly |
| ZA17 | Group E | Unclassified | 11465 | 1899228 | 1910693 | Sheath, tail tube, tape measure, baseplate, tail fiber, tail fiber assembly |
| NY26 | Group F | Unclassified | 1262 | 1044035 | 1045297 | Terminase large, terminase small |
| ZA17 | Group F | Unclassified | 1262 | 974893 | 976155 | Terminase small, terminase large |
| A2C | Group G | Potential partial Podoviridae | 17340 | 1652408 | 1669748 | Capsid and scaffold |
| AS3 | Group G | Potential partial Podoviridae | 17338 | 1708189 | 1725527 | Capsid and scaffold |
| NY26 | Group G | Potential partial Podoviridae | 7333 | 927590 | 934923 | Capsid and scaffold |
| NY26 | Group G | Potential partial Podoviridae | 4725 | 904118 | 908843 | No structural component genes present |
| ZA17 | Group G | Potential partial Podoviridae | 9435 | 1918369 | 1927804 | Capsid and scaffold |
| ZA17 | Group G | Potential partial Podoviridae | 4415 | 1894812 | 1899227 | No structural component genes present |
| A2C | Group H | Potential partial Podoviridae | 23791 | 1765100 | 1788891 | Capsid and scaffold |
| A2C | Group H | Potential partial Podoviridae | 3995 | 1353793 | 1357788 | No structural component genes present |
| AS3 | Group H | Potential partial Podoviridae | 23791 | 1820872 | 1844663 | Capsid and scaffold |
| AS3 | Group H | Potential partial Podoviridae | 3995 | 1369697 | 1373692 | No structural component genes present |
| NY26 | Group H | Potential partial Podoviridae | 18800 | 1440751 | 1459551 | Capsid and scaffold |
| NY26 | Group H | Potential partial Podoviridae | 7739 | 1838889 | 1846628 | No structural component genes present |
| ZA17 | Group H | Potential partial Podoviridae | 8009 | 1785957 | 1793966 | Capsid and scaffold |
| ZA17 | Group H | Potential partial Podoviridae | 4530 | 2042941 | 2047471 | No structural component genes present |
| ZA17 | Group H | Potential partial Podoviridae | 3934 | 1320786 | 1324720 | No structural component genes present |
| NY26 | Group I | Unclassified | 4064 | 923517 | 927581 | No structural component genes present |
| ZA17 | Group I | Unclassified | 3827 | 1914492 | 1918319 | No structural component genes present |
| A2C | Group K | Unclassified | 8112 | 1743480 | 1751592 | Tail fiber |
| AS3 | Group K | Unclassified | 8112 | 1799254 | 1807366 | Tail fiber |
| NY26 | Group K | Unclassified | 4468 | 1823621 | 1828089 | No structural component genes present |
| NY26 | Group K | Unclassified | 1912 | 1860300 | 1862212 | No structural component genes present |
| ZA17 | Group K | Unclassified | 6945 | 1814622 | 1821567 | Tail fiber |
| A2C | Group L | Partial Myoviridae of Type 1 Cluster 8, Mu-like | 9347 | 1888948 | 1898295 | Base plate, major capsid, baseplate assembly, baseplate assembly, baseplate assembly, tail fiber, tail fiber assembly |
| AS3 | Group L | Partial Myoviridae of Type 1 Cluster 8, Mu-like | 9346 | 1944714 | 1954060 | Base plate, major capsid, baseplate assembly, baseplate assembly, baseplate assembly, tail fiber, tail fiber, tail fiber assembly |
| NY26 | Group L | Partial Myoviridae of Type 1 Cluster 8, Mu-like | 10691 | 2007716 | 2018407 | Portal, baseplate, baseplate assembly, baseplate assembly, tail fiber, tail fiber |
| NY26 | Group L | Partial Myoviridae of Type 1 Cluster 8, Mu-like | 1462 | 2003770 | 2005232 | Tail fiber, tail fiber assembly |
| ZA17 | Group L | Partial Myoviridae of Type 1 Cluster 8, Mu-like | 10664 | 1730683 | 1741347 | portal, capsid, baseplate assembly, baseplate assembly, baseplate assembly, tail fiber |
| NY26 | Group M | Potential partial Podoviridae | 4065 | 563073 | 567138 | Capsid and scaffold |
| ZA17 | Group M | Potential partial Podoviridae | 4016 | 460289 | 464305 | Capsid and scaffold |
| A2C | Group N | Unclassified | 1037 | 866284 | 867321 | Base plate |
| AS3 | Group N | Unclassified | 1036 | 880679 | 881715 | Base plate |
| NY26 | Group N | Unclassified | 1031 | 1073583 | 1074614 | Base plate |
| ZA17 | Group N | Unclassified | 1029 | 938593 | 939622 | Base plate |
| NY26 | Group O | Unclassified | 1474 | 1144079 | 1145553 | Tail fiber |
| ZA17 | Group O | Unclassified | 1473 | 1196666 | 1198139 | Tail fiber |
| NY26 | Group P | Potential Myoviridae Type 1 cluster 3 or closely related | 11919 | 1328826 | 1340745 | Terminase small, terminase large, portal, major capsid |
| A2C | Group Q | Unclassified | 512 | 762160 | 762672 | Terminase large, terminase large |
| AS3 | Group Q | Unclassified | 512 | 776559 | 777071 | Terminase large, terminase large |
| NY26 | Group Q | Unclassified | 513 | 663886 | 664399 | Terminase large, terminase large |
| ZA17 | Group Q | Unclassified | 92 | 810930 | 811022 | Terminase large |
| NY26 | Group R | Unclassified | 4842 | 1896813 | 1901655 | No structural component genes present |
| A2C | Group S | Unclassified | 605 | 1602190 | 1602795 | No structural component genes present |
| AS3 | Group S | Unclassified | 605 | 1657985 | 1658590 | No structural component genes present |
| NY26 | Group S | Unclassified | 1446 | 254020 | 255466 | No structural component genes present |
| ZA17 | Group S | Unclassified | 1446 | 1657231 | 1658677 | No structural component genes present |
| A2C | Group T | Unclassified | 263 | 1604202 | 1604465 | No structural component genes present |
| AS3 | Group T | Unclassified | 263 | 1659997 | 1660260 | No structural component genes present |
| NY26 | Group T | Unclassified | 266 | 256872 | 257138 | No structural component genes present |
| ZA17 | Group T | Unclassified | 263 | 1660083 | 1660346 | No structural component genes present |
| AS3 | APSE-3 | Podoviridae Type 3 | 38991 | 1430908 | 1469899 | Terminase large, portal, capsid scaffold, major capsid, tail fiber, tail fiber assembly |
| ZA17 | APSE-8 | Podoviridae Type 3 | 39144 | 1226217 | 1265361 | Terminase large, portal, capsid scaffold, major capsid, tail fiber, tail fiber assembly |
| NY26 | APSE-2 | Podoviridae Type 3 | 39883 | 1514811 | 1554694 | Terminase large, portal, capsid scaffold, major capsid, tail fiber, tail fiber assembly |
