# Supplementary material for: Culture-Facilitated Comparative Genomics of the Facultative Symbiont Hamiltonella defensa
Source: Genome Biol Evol. 2018 Feb 14;10(3):786–802. doi: 10.1093/gbe/evy036 (PMC5841374; doi:10.1093/gbe/evy036)
Supplement: Supplementary Data [file evy036_supp.zip › Table-S4.docx]

**Table S4**

Classification and Gene Inventory for the Plasmid Islands in the A2C, AS3, NY26 and ZA17 Strains of *H. defensa*. Plasmid Islands for 5AT are Summarized by Degnan et al. (2009).

| **Strain** | **Group** | **Classification** | **Lenght** | **Start** | **Stop** | **Structural Marker Gene inventroy** |
| --- | --- | --- | --- | --- | --- | --- |
| A2C | Group A | pHD5AT/pHDAS3.1/pHDA2C.1/pHDA2C.3 | 42790 | 1604516 | 1647306 | PilV, VapB antitoxin, VapC toxin, DNA binding protein, FinO, TrbC, TrbB, TrbA, Surface exclusion protein, TraY, TraX, TraW, TraU, TraQ, TraP, TraN, TraM, TraL, TraC, Conjugal transfer protein, Virulence plasmid protein, PilT, TraH, PilM, PilV, Phd antitoxin, Doc toxin, PulF, PilB, PilO, Lipoprotein |
| AS3 | Group A | pHD5AT/pHDAS3.1/pHDA2C.1/pHDA2C.3 | 42775 | 1660311 | 1703086 | PilV, VapB antitoxin, VapC toxin, DNA binding protein, FinO, TrbC, TrbB, TrbA, Surface exclusion protein, TraY, TraX, TraW, TraU, TraQ, TraP, TraN, TraM, TraL, TraC, Conjugal transfer protein, Virulence plasmid protein, PilT, TraH, PilM, PilV, Phd antitoxin, Doc toxin, PulF, PilB, PilO, Lipoprotein |
| ZA17 | Group A | pHD5AT/pHDAS3.1/pHDA2C.1/pHDA2C.3 | 43284 | 1660337 | 1703621 | PilV, VapB antitoxin, VapC toxin, DNA binding protein, FinO, TrbC, TrbB, TrbA, Surface exclusion protein, TraY, TraX, TraW, TraU, Plasmid stabilization protein, TraQ, TraP, TraO, TraN, TraM, TraL, TraC, Conjugal transfer protein, Virulence plasmid protein, PilT, TraH, PilM, PilV, Phd antitoxin, Doc toxin, PulF, PilB, PilO, Lipoprotein |
| NY26 | Group A | pHD5AT/pHDAS3.1/pHDA2C.1/pHDA2C.3 | 4530 | 257146 | 261676 | PilV, VapB antitoxin, VapC toxin, DNA binding protein |
| NY26 | Group A | pHD5AT/pHDAS3.1/pHDA2C.1/pHDA2C.3 | 33544 | 942056 | 975600 | VapB antitoxin, VapC toxin, TrbC, TrbB, TrbA, Surface exclusion protein, TraY, TraX, TraW, TraU, Plasmid stabilization protein, TraQ, TraP, TraO, TraN, TraM, TraL, TraC, Conjugal transfer protein, Virulence plasmid protein, PilT, TraH, PilM, PilV, Phd antitoxin, Doc toxin, PulF, PilB, PilO, Lipoprotein |
| A2C | Group B | pHDZA17.2 | 35421 | 1055837 | 1091258 | VirB4, VirB5, TraG, VirB6, VirB8, VirB9, VirB10, VirB11, VirD4, PilM, PilN, PilO, PilQ, small heat shock protein, PulF, PilT, PilV, StdB, Plasmid replication initiation protein |
| AS3 | Group B | pHDZA17.2 | 35421 | 1071772 | 1107193 | VirB4, VirB5, TraG, VirB6, VirB8, VirB9, VirB10, VirB11, VirD4, PilM, PilN, PilO, PilQ, small heat shock protein, PulF, PilT, PilV, StdB, Plasmid replication initiation protein |
| NY26 | Group B | pHDZA17.2 | 28472 | 1241450 | 1269922 | DNA-3-methyladenine glycosylase II, helix-turn-helix transcriptional regulator, VirB1, VirB4, VirB5, TraG, VirB6, VirB8, VirB9, VirB10, VirB11, VirD4, PilM, PilN, PilO, PilQ, small heat shock protein, PulF, PilT, PilV, StdB, Plasmid replication initiation protein |
| A2C | Group C | pHDZA17.3 | 2493 | 1047754 | 1050247 | Transcription regulator |
| AS3 | Group C | pHDZA17.3 | 2493 | 1063691 | 1066184 | Transcription regulator |
| ZA17 | Group C | pHDZA17.3 | 8365 | 984742 | 993106 | Transcription regulator, DNA-damage-inducible protein J |
| NY26 | Group C | pHDZA17.3 | 8354 | 1053819 | 1062172 | Transcription regulator, DNA-damage-inducible protein J |
| A2C | Group D | pHDZA17.2 | 18657 | 1028701 | 1047358 | DNA-3-methyladenine glycosylase II, TraR, RepB, PilQ, Inner membrane protein, VapB, DNA primase |
| AS3 | Group D | pHDZA17.2 | 9096 | 1054199 | 1063295 | DNA-3-methyladenine glycosylase II, TraR, RepB, Phage DNA primase, PilQ, Inner membrane protein, VapB, DNA primase |
| ZA17 | Group D | pHDZA17.2 | 4465 | 1137604 | 1142069 | RepB, Phage DNA primase, PilQ, Inner membrane protein, VapB, DNA primase |
| NY26 | Group D | pHDZA17.2 | 4467 | 1270065 | 1274532 | RepB, Phage DNA primase, PilQ, Inner membrane protein, VapB, DNA primase |
| AS3 | Group E | pHDA2C.2/pHDAS3.2/pHDZA17.1 | 21018 | 1032183 | 1053201 | TraN, TraU, TraW, TrhF, TraC, Disulfide isomerase, TraA, TraB, TraK, TraE, TraL, TraD, Tral, DNA binding protein |
| ZA17 | Group F | pHDZA17.1 | 8131 | 1120074 | 1128205 | Cytosine permease, helix-turn-helix transcriptional regulator, transcriptional regulator |
| NY26 | Group G | pHD5AT/pHDAS3.1/pHDA2C.1/pHDA2C.3 | 43049 | 1284700 | 1327749 | N-acetyltransferase, MutL, RTX toxin, Programmed cell death toxin ChpB, Programmed cell death antitoxin MazE, ParA, ParG, MsrB, RTX toxin, Mobilization protein, DNA binding, FinO, Conjugal transfer protein, PilT, Tral, PilM, PilV, PilR, General secretion pathway E, RelE toxin, XRE transcriptional regulator, R64 PilN family, PilL |
| ZA17 | Group H | pHDZA17.2 | 6300 | 1199517 | 1205817 | PilQ, Small heat shock protein, PulF, PilT, YafQ toxin, DNA damage-inducible |
| NY26 | Group H | pHDZA17.2 | 5495 | 1137100 | 1142595 | PilQ, Small heat shock protein, PulF, PilT, YafQ toxin, DNA damage-inducible |
| NY26 | Group I | pHDA2C.3/pHDAS3.1 | 15603 | 725471 | 741074 | Glycerol dehydrogenase, FinO, DNA binding protein, Mobilization protein, ParA, HicA, HicB, Signal peptidase, Transmembrane protein |
| A2C | Group J | pPSR1 from Pseudomonas syringae | 17539 | 1113000 | 1130539 | VirB4, VirB5, VagC, VapC toxin, VirB6, Plasmid stability-like, VapB antitoxin, StdB, VirB8, VirB9, Conjugal transfer, VirB11, VirD4, KikA, PilM |
| AS3 | Group J | pPSR1 from Pseudomonas syringae | 17535 | 1128933 | 1146468 | VirB4, VirB5, VagC, VapC toxin, VirB6, Plasmid stability-like, VapB antitoxin, StdB, VirB8, VirB9, Conjugal transfer, VirB11, VirD4, KikA, PilM |
| ZA17 | Group J | pPSR1 from Pseudomonas syringae | 11460 | 1161508 | 1172968 | VirB4, VirB5, VagC, VapC toxin, VirB6, Plasmid stability-like, VapB antitoxin, StdB, VirB8, VirD4, KikA, PilM |
| NY26 | Group J | pPSR1 from Pseudomonas syringae | 14438 | 416708 | 431146 | VirB4, VirB5, VagC, VapC toxin, VirB6, Plasmid stability-like, VapB antitoxin, StdB, VirB8, VirB9, Conjugal transfer, VirB11, VirD4 |
| A2C | Group K | 5 Degnan et al 2009 | 40989 | 885330 | 926319 | tRNA GTPase, RelE / StbE toxin, RelB / StbD antitoxin, Transcriptional regulator, Beta galactosidase, MobA, Replication protein, preQ0 reductase, preQ0 transporter, HicA related, ParA, MobC, Tral related, RfbD, RfbE, Glycosyltransferase, RepA1, MutL, R64 PilN family |
| AS3 | Group K | 5 Degnan et al 2009 | 40987 | 899707 | 940694 | tRNA GTPase, RelE / StbE toxin, RelB / StbD antitoxin, Transcriptional regulator, Beta galactosidase, MobA, Replication protein, preQ0 reductase, preQ0 transporter, HicA related, ParA, MobC, Tral related, RfbD, RfbE, Glycosyltransferase, RepA1, MutL, R64 PilN family |
| ZA17 | Group K | 5 Degnan et al 2009 | 31487 | 942169 | 973656 | YoeB toxin, YefM antitoxin, Type IIS RE, Restriction enzyme Bcgl, tRNA GTPase, RelE / StbE toxin, RelB / StbD antitoxin, Transcriptional regulator, Beta galactosidase, MobA, Replication protein, preQ0 reductase, preQ0 transporter, HicA related, ParA, MobC, Tral related, RepA1, Pentapeptide repeat, R64 PilN family |
| NY26 | Group K | 5 Degnan et al 2009 | 39845 | 1002938 | 1042783 | YoeB toxin, YefM antitoxin, Type IIS RE, Restriction enzyme Bcgl, tRNA GTPase, RelE / StbE toxin, RelB / StbD antitoxin, Transcriptional regulator, Beta galactosidase, MobA, Replication protein, preQ0 reductase, preQ0 transporter, HicA related, ParA, MobC, Tral related |
| A2C | Group L | 9 Degnan et al 2009 | 25415 | 1863447 | 1888862 | ParA, RTX toxin, Acetyltransferase, VapC toxin, VapB antitoxin, DNA primase, Inner membrane protein, Dca, MobC, Mobilization protein, DNA relaxase Tral |
| AS3 | Group L | 9 Degnan et al 2009 | 25413 | 1919215 | 1944628 | ParA, RTX toxin, Acetyltransferase, VapC toxin, VapB antitoxin, DNA primase, Inner membrane protein, Dca, MobC, Mobilization protein, DNA relaxase Tral |
| ZA17 | Group L | 9 Degnan et al 2009 | 24915 | 1705549 | 1730464 | ParA, RTX toxin, Acetyltransferase, VapC toxin, VapB antitoxin, DNA primase, Inner membrane protein, Dca, MobC, Mobilization protein, DNA relaxase Tral |
| NY26 | Group L | 9 Degnan et al 2009 | 26974 | 1976710 | 2003684 | ParA, RTX toxin, Acetyltransferase, VapC toxin, VapB antitoxin, DNA primase, Inner membrane protein, Dca, MobC, Mobilization protein, DNA relaxase Tral |
| NY26 | Group L | 9 Degnan et al 2009 | 1439 | 2006185 | 2007624 | DNA relaxase Tral |
