# Supplementary material for: Culture-Facilitated Comparative Genomics of the Facultative Symbiont Hamiltonella defensa
Source: Genome Biol Evol. 2018 Feb 14;10(3):786–802. doi: 10.1093/gbe/evy036 (PMC5841374; doi:10.1093/gbe/evy036)
Supplement: Supplementary Data [file evy036_supp.zip › Table-S5.docx]

**Table S5**

Mean dN and dS values for between strain comparisons of aphid and whitefly-associated *H. defensa*.

| **Strain 1** | **Strain 2** | **Mean-dn** | **Mean-ds** | **Orthologs** |
| --- | --- | --- | --- | --- |
| 5AT | NY26 | 0.0004 | 0.0038 | 1408 |
| A2C | AS3 | 0.0006 | 0.0006 | 1379 |
| A2C | 5AT | 0.0152 | 0.0354 | 1284 |
| A2C | NY26 | 0.0096 | 0.0236 | 1282 |
| AS3 | 5AT | 0.0116 | 0.0353 | 1333 |
| 5AT | ZA17 | 0.0167 | 0.0468 | 1348 |
| ZA17 | NY26 | 0.0116 | 0.0347 | 1350 |
| A2C | ZA17 | 0.0119 | 0.0337 | 1313 |
| AS3 | ZA17 | 0.0124 | 0.0349 | 1362 |
| MEAM1 | MED | 0.0012 | 0.0021 | 1094 |
| MEAM1 | A2C | 0.0252 | 0.0934 | 1041 |
| MED | A2C | 0.0232 | 0.0937 | 1067 |
| AS3 | MEAM1 | 0.0269 | 0.1011 | 1082 |
| AS3 | MED | 0.0248 | 0.1006 | 1111 |
| 5AT | MEAM1 | 0.0266 | 0.1008 | 1072 |
| MEAM1 | NY26 | 0.0267 | 0.0995 | 1068 |
| MEAM1 | ZA17 | 0.0236 | 0.0883 | 1076 |
| 5AT | MED | 0.0257 | 0.1120 | 1095 |
| MED | NY26 | 0.0256 | 0.1109 | 1094 |
| MED | ZA17 | 0.0250 | 0.0993 | 1108 |
