# Supplementary material for: Culture-Facilitated Comparative Genomics of the Facultative Symbiont Hamiltonella defensa
Source: Genome Biol Evol. 2018 Feb 14;10(3):786–802. doi: 10.1093/gbe/evy036 (PMC5841374; doi:10.1093/gbe/evy036)
Supplement: Supplementary Data [file evy036_supp.zip › Table-S6.docx]

**Table S6**

Summary of RNAseq data

| A2C | Replicate 1 | | Replicate 2 | |  |  |
| --- | --- | --- | --- | --- | --- | --- |
|  | Reads | Percentage | Reads | Percentage |  |  |
| A2C Chromosome | 23,177,076 | 91,17% | 26,440,952 | 91,36% |  |  |
| pHDA2C.1 | 1,409,106 | 5,54% | 1,395,000 | 4,82% |  |  |
| pHDA2C.2 | 146,690 | 0,58% | 136,992 | 0,47% |  |  |
| pHDA2C.3 | 302,460 | 1,19% | 359,530 | 1,24% |  |  |
| Unmapped | 386,200 | 1,52% | 609,840 | 2,11% |  |  |
| Total | 25,421,532 | 100% | 28,942,314 | 100% |  |  |
|  |  |  |  |  |  |  |
| AS3 | Replicate 1 | | Replicate 2 | | Replicate 3 | |
|  | Reads | Percentage | Reads | Percentage | Reads | Percentage |
| AS3 Chromosome | 20,255,264 | 91,81% | 25,395,258 | 91,05% | 27,467,858 | 92,15% |
| pHDAS3.1 | 1,221,930 | 5,54% | 1,675,344 | 6,01% | 1,566,394 | 5,26% |
| pHDAS3.2 | 136,168 | 0,62% | 138,544 | 0,50% | 177,896 | 0,60% |
| Unmapped | 448,392 | 2,03% | 683,046 | 2,45% | 594,856 | 2,00% |
| Total | 22,061,754 | 100% | 27,892,192 | 100% | 29,807,004 | 100% |
|  |  |  |  |  |  |  |
| ZA17 | Replicate 1 | | Replicate 2 | | Replicate 3 | |
|  | Reads | Percentage | Reads | Percentage | Reads | Percentage |
| ZA17 Chromosome | 27,319,712 | 94,57% | 36,443,212 | 95,15% | 46,851,826 | 93,84% |
| pHDZA17.1 | 513,994 | 1,78% | 576,050 | 1,50% | 1,282,346 | 2,57% |
| pHDZA17.2 | 126,202 | 0,44% | 175,904 | 0,46% | 252,986 | 0,51% |
| pHDZA17.3 | 236,130 | 0,82% | 202,494 | 0,53% | 368,766 | 0,74% |
| Unmapped | 691,862 | 2,39% | 904,364 | 2,36% | 1,168,900 | 2,34% |
| Total | 28,887,900 | 100% | 38,302,024 | 100% | 49,924,824 | 100% |
