# Supplementary material for: Culture-Facilitated Comparative Genomics of the Facultative Symbiont Hamiltonella defensa
Source: Genome Biol Evol. 2018 Feb 14;10(3):786–802. doi: 10.1093/gbe/evy036 (PMC5841374; doi:10.1093/gbe/evy036)
Supplement: Supplementary Data [file evy036_supp.zip › Table-S7.docx]

**Table S7**

Base modifications identified in the A2C, AS3, ZA17 and NY26 strains of *H. defensa* by SMRT sequencing.

| **Strain** | **Sequence motif** | | | | | |
| --- | --- | --- | --- | --- | --- | --- |
|  | G**^m6^A**TC | | GC**^m6^A**N_6_TCC | | CG**^m6^A**N_6_TCC | |
|  | No. of motif | % modified motif | No. of motif | % modified motif | No. of motif | % modified motif |
| A2C | 16980 | 93,7% | 571 | 0,0% | 542 | 0,0% |
| AS3 | 17262 | 0,0% | 582 | 0,0% | 550 | 0,0% |
| ZA17 | 17463 | 0,3% | 576 | 91,1% | 527 | 0,1% |
| NY26 | 16412 | 94,8% | 531 | 0,2% | 576 | 71,0% |
