# Supplementary material for: Culture-Facilitated Comparative Genomics of the Facultative Symbiont Hamiltonella defensa
Source: Genome Biol Evol. 2018 Feb 14;10(3):786–802. doi: 10.1093/gbe/evy036 (PMC5841374; doi:10.1093/gbe/evy036)
Supplement: Supplementary Data [file evy036_supp.zip › Table-S8.docx]

**Table S8**

| Loci identified in different strains of *H. defensa* with potential roles in restriction modification. | | | | |  |  |  |  |  |
| --- | --- | --- | --- | --- | --- | --- | --- | --- | --- |
|  | Locus 1 | Locus 2 | Locus 3 | Locus 4 | Locus 5 | Locus 6 | Locus 7 | Locus 8 | Locus 9 |
| Function | Type I MTase and REase | Orphan MTase | Type II MTase | Type II MTase | Type II MTase | Type II MTase | Type II MTase + REase + Mismatch repair (Nicking) | Type IIG MTase and REase + Specifity domain | Type II MTase |
| Modified base | ^m6^A | ^m6^A | ^m5^C | ^m4^C or ^m6^A | ^m4^C or ^m6^A | ^m5^C | ^m5^C | ^m6^A | ^m5^C |
| Modified motif | Unknown | GATC | Unknown | Unknown | Unknown | Unknown | Unknown | Unknown | Unknown |
| **A2C** | **Pseudo*** | **Yes** | **Yes** | **Yes** | **Pseudo^t^** | **No** | **No + No + No** | **No + No** | **No** |
|  | BJP41_07495 | BJP41_07780 | BJP41_07955 | BJP41_08130 | BJP41_08345 | / | / + / + / | / + / | / + / |
| **AS3** | **Pseudo*** | **Pseudo*** | **Yes** | **Yes** | **Pseudo^t^** | **No** | **No + No + No** | **No + No** | **No** |
|  | BJP42_07555 | BJP42_07855 | BJP42_08225 | BJP42_08400 | BJP42_08620 | / | / + / + / | / + / | / + / |
| **ZA17** | **Yes** | **Pseudo*** | **Yes** | **Yes** | **Yes** | **Yes** | **Yes + Yes + Yes** | **Yes** | **Yes** |
|  | BJP43_07310 | BJP43_07060 | BJP43_06665 | BJP43_01340 | BJP43_08385 | BJP43_01510 | BJP43_04715 + BJP43_04720 + BJP43_04725 | BJP43_05015 + BJP43_05020 | BJP43_10650 |
| **NY26** | **Yes** | **Yes** | **No** | **Yes** | **No** | **No** | **Pseudo* + Yes + Yes** | **Yes** | **Yes** |
|  | BJP44_08270 | BJP44_08545 | / | BJP44_05260 | / | / | BJP44_06020 + BJP44_06015 + BJP44_06010 | BJP44_05600 + BJP44_05605 | BJP44_04715 |
| **5AT** | **Yes** | **Yes** | **No** | **Yes** | **No** | **No** | **Pseudo* + Yes + Yes** | **Yes** | **Yes** |
|  | 5AT_08270 | 5AT_08545 | / | 5AT_05260 | / | / | 5AT_06020 + 5AT_06015 + 5AT_06010 | 5AT_05600 + 5AT_05605 | 5AT_04715 |
| Region | Phage Island group H | Main chromosome | Phage Island group A | Phage Island group B, C and D | Main chromosome | Phage Island group D | Main chromosome | Plasmid Island group K | Phage Island group E |
| Database | BlastP, Hmmer and Pfam | REBASE | REBASE | REBASE | REBASE | REBASE | REBASE | REBASE | REBASE |
| + separate different genes from the same locus; * Pseudogene due to point mutation; t Pseudogene due to TE transposition. | | | | |  |  |  |  |  |
