# Supplementary figures and images for: Culture-Facilitated Comparative Genomics of the Facultative Symbiont Hamiltonella defensa
Source: Genome Biol Evol. 2018 Feb 14;10(3):786–802. doi: 10.1093/gbe/evy036 (PMC5841374; doi:10.1093/gbe/evy036)

Fig. S1

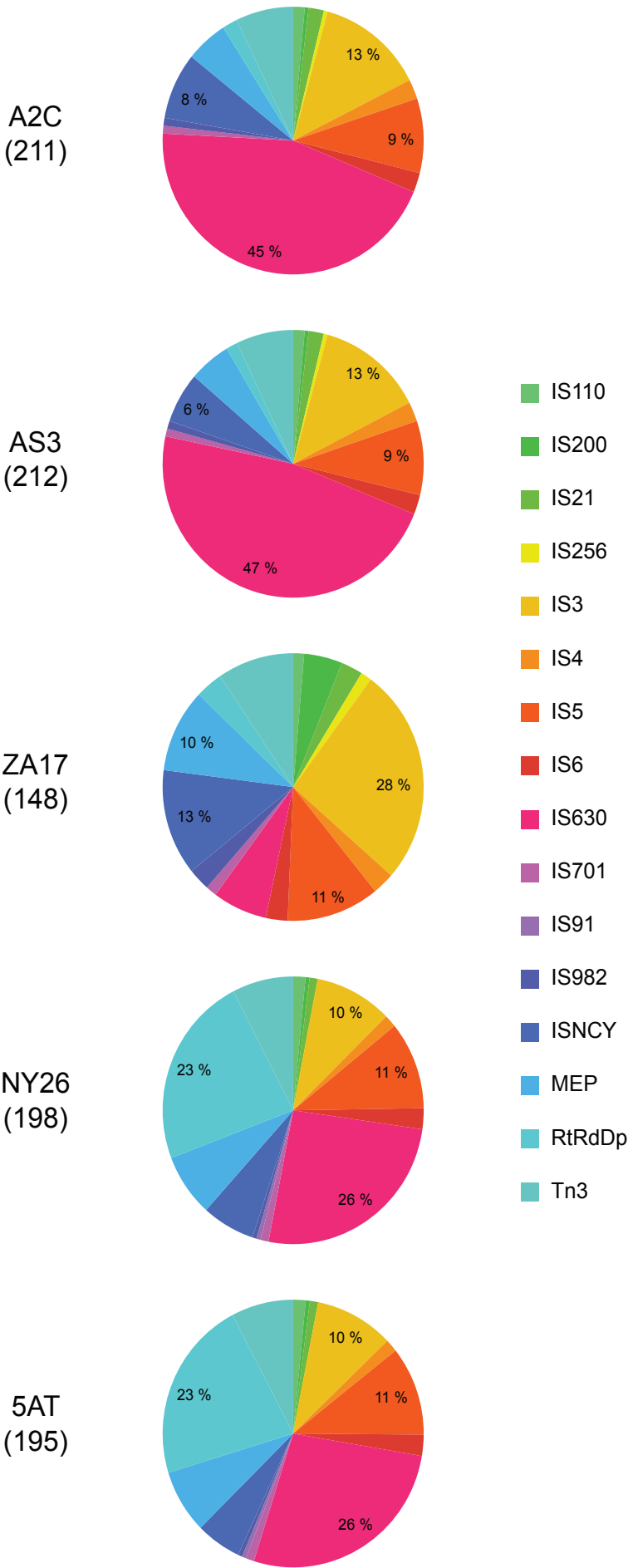

Supplement: Supplementary Data [file evy036_supp.zip › Fig_S1.pdf]

Fig. S2

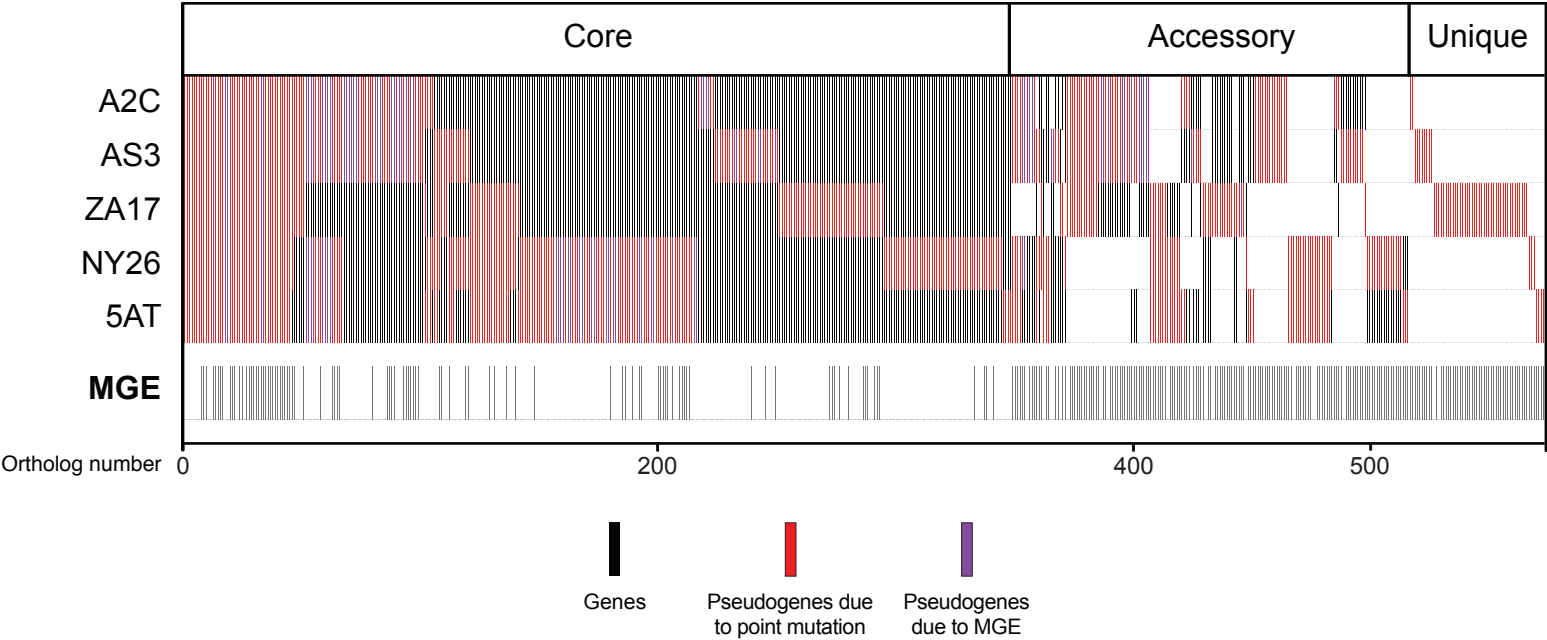

Supplement: Supplementary Data [file evy036_supp.zip › Fig_S2.pdf]
