# Supplementary material for: Culture-Facilitated Comparative Genomics of the Facultative Symbiont Hamiltonella defensa
Source: Genome Biol Evol. 2018 Feb 14;10(3):786–802. doi: 10.1093/gbe/evy036 (PMC5841374; doi:10.1093/gbe/evy036)
Supplement: Supplementary Data [file Fig_S3.pdf › Fig_S3.pdf]

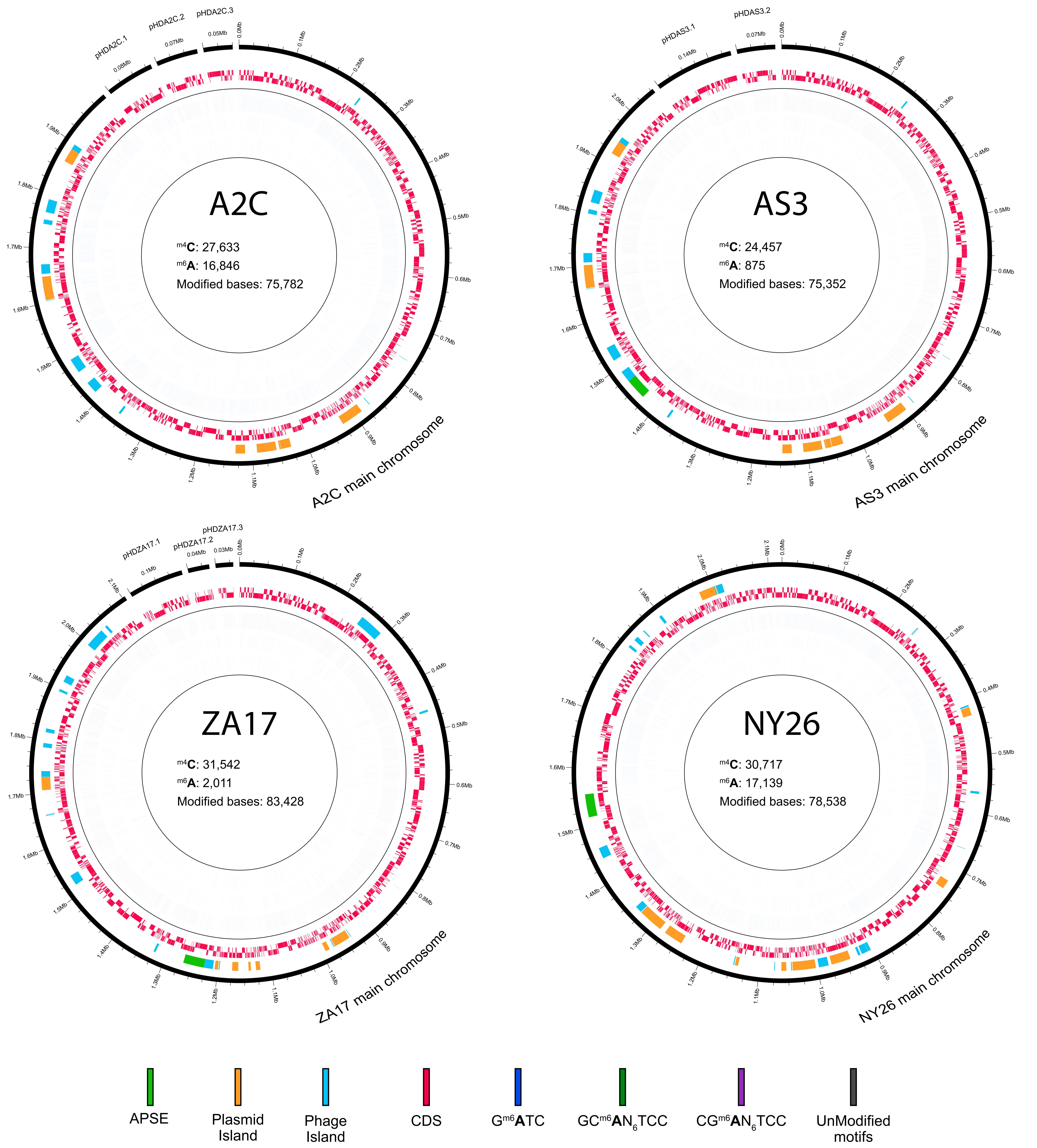

pHDA2C.1
pHDA2C.2
pHDA2C.3
A2C
m4C: 27,633
m6A: 16,846
Modified bases: 75,782
A2C main chromosome
pHDAS3.1
pHDAS3.2
AS3
m4C: 24,457
m6A: 875
Modified bases: 75,352
AS3 main chromosome
pHDZA17.1
pHDZA17.2
pHDZA17.3
ZA17
m4C: 31,542
m6A: 2,011
Modified bases: 83,428
ZA17 main chromosome
NY26
m4C: 30,717
m6A: 17,139
Modified bases: 78,538
NY26 main chromosome
APSE
Plasmid Island
Phage Island
CDS
Gm6ATC
GCm6AN6TCC
CGm6AN6TCC
UnModified motifs
